# PLEKHG5 regulates autophagy, survival and MGMT expression in U251-MG glioblastoma cells

**DOI:** 10.1038/s41598-020-77958-3

**Published:** 2020-12-14

**Authors:** Kaya Elisa Witte, Carsten Slotta, Melanie Lütkemeyer, Angelika Kitke, Roland Coras, Matthias Simon, Christian Kaltschmidt, Barbara Kaltschmidt

**Affiliations:** 1 Department of Cell Biology, University of Bielefeld, Universitätsstr. 25, 33615 Bielefeld, Germany; 2 Molecular Neurobiology, University of Bielefeld, Universitätsstr. 25, 33615 Bielefeld, Germany; 3 Department of Neuropathology, University Hospital Erlangen, Schwabachanlage 6, 91054 Erlangen, Germany; 4 Department of Neurosurgery, Protestant Hospital of Bethel Foundation, Burgsteig 13, 33617 Bielefeld, Germany; 5 Research Association of BioMedicine Bielefeld, FBMB, Maraweg 21, 33617 Bielefeld, Germany

**Keywords:** Cancer, Cell biology, Molecular biology, Diseases, Oncology, Diseases of the nervous system

## Abstract

A signalling pathway involving PLEKHG5 (guanine exchange factor) for the Ras superfamily member RAB26 to transcription factor NF-κB was discovered in autophagy. PLEKHG5 was reported in glioblastoma multiforme (GBM) and correlates with patient survival. Thus, the generation of a cellular model for understanding PLEKHG5 signalling is the study purpose. We generated a CRISPR/Cas9-mediated knockout of PLEKHG5 in U251-MG glioblastoma cells and analysed resulting changes. Next, we used a mRFP-GFP-LC3^+^ reporter for visualisation of autophagic defects and rescued the phenotype of *PLEKHG5* wildtype via transduction of a constitutively active RAB26QL-plasmid. Effects of overexpressing RAB26 were investigated and correlated with the O^6^-methylguanine-DNA methyltransferase (MGMT) and cellular survival. *PLEKHG5* knockout showed changes in morphology, loss of filopodia and higher population doubling times. Accumulation of autolysosomes was resulted by decreased LAMP-1 in PLEKHG5-deficient cells. Rescue of *PLEKHG5*^−/−^ restored the downregulation of RhoA activity, showed faster response to tumour necrosis factor and better cellular fitness. MGMT expression was activated after RAB26 overexpression compared to non-transduced cells. Survival of *PLEKHG5* knockout was rescued together with sensitivity to temozolomide by RAB26QL. This study provides new insights in the PLEKHG5/RAB26 signalling within U251-MG cells, which suggests potential therapeutic strategies in other glioma cells and further in primary GBM.

## Introduction

Glioblastoma multiforme (GBM) is the most frequent malignant primary human brain tumour in adults^1^. Despite radiation and temozolomide (TMZ) chemotherapy, the average survival time of GBM patients is no longer than 15 months^2^. A frequently used model of glioblastoma is the cell line U251-MG, which was derived from a grade III-IV astrocytoma isolated from a male patient in 1973^3,4^. Furthermore, U251-MG cells are commonly used for examining the role of various genes in tumour pathogenesis^5^ and the efficacy of therapeutic agents^6^.

Recently, Pleckstrin homology containing family member 5 (PLEKHG5) has been described as a prognostic biomarker in glioblastoma patients^7^. PLEKHG5 is a guanine exchange factor (GEF), which is highly expressed in endothelial cells of the nervous system and in different cancer cells^5,8,9^. PLEKHG5 functions as a GEF for Rho GTPases, which are key regulators for cellular dynamics. Activation of Rho by binding a GEF like PLEKHG5, was also described to be involved in polarity-orientated cell migration in U251-MG cells^5^. Thus, Dachsel and colleagues published that the signalling via the PLEKHG5/RhoA pathway is a major contributor in the dissemination and poor outcome of GBM.

Direct expression of PLEKHG5 has been shown to activate nuclear factor “kappa-light-chain-enhancer” in activated B-cells (NF-κB) signalling in different cell lines in vitro as well as in spinal cord and brain in vivo^10^. RhoA was also discovered to be an activator of the transcription factor NF-κB^11^. These findings provide an additional link between cancer and PLEKHG5 signalling, since NF-κB is a key player in cancer development and progression^12,13^. It also plays a major role in various cellular functions like inflammation, immune response, apoptosis and proliferation^11,12,14^, particularly in the nervous system^15,16^. Of note, an activation of the NF-κB transcription factor is frequently observed in GBM^17^. Soubannier and Stifani reported that the canonical NF-κB activation results from previous stimulation with cytokines like tumour necrosis factor alpha (TNFα) in GBM. Overexpression of TNFα is known to decrease cellular fitness, inhibit proliferative behaviour and promote apoptosis in U251-MG cells^18^.

We recently reported, that autophagy-mediated clearance of synaptic vesicles is regulated by PLEKHG5 together with the small GTPase RAB26 and identified PLEKHG5 as a specific GEF for RAB26^9^. In this context, a constitutive active variant of RAB26 rescued the autophagic pathway in PLEKHG5-deficient mice^9,11^. Of note, RAB26 was predominantly associated and described to be responsible for coordination of lysosome traffic, specifically via the lysosomal-associated membrane protein-1 (LAMP-1), which is a main component of the autophagic signalling^19^. Generally, autophagy is characterised by initial formation of autophagosome followed by autophagosome-lysosome fusion and ultimately lysosomal enzyme degradation to ensure cellular viability and organelle renewal^20^. Blocking any of the above steps leads to a decline in autophagy flux. Prior to the formation of autophagosome, an induction signal leads to the processing of a phagophore, loaded with microtubule-associated proteins 1A/1B light chain 3B (LC3) on the membrane surface. For degradation, the autophagosome is fused with a lysosome resulting in the formation of an autolysosome. Lysosomal hydrolytes degrade the cargo, while autophagic-derived nutrients are recycled^21^. Two major proteins are estimated to contribute for about 50% of the lysosomal membrane, namely LAMP-1 and LAMP-2. Both are thought to protect the lysosomal membrane against hydrolytic enzymes within the lysosome. The presence of LAMP proteins is one of the major definitions of the lysosomal compartment^22^. Notably, the expression of both glycoproteins is linked to each other, which results in a LAMP-1/LAMP-2-homeostasis. Interestingly, LAMP-1 and LAMP-2 expression has been observed in various tumours and has been shown to correlate with a high metastatic potential^23,24^, whereas *LAMP-1* was also published to be overexpressed in some glioblastomas^25^. In context with cancer, autophagy in general also plays a critical role in the survival of cancerogenic cells undergoing starvation due to deficiencies in nutrition supply within the tumour microenvironment^26^. Here, specifically glioblastomas might be unusually dependent on autophagy^26^, which might be one of the mechanisms underlying chemoresistance in GBM^27^.

The chemotherapeutic agent temozolomide (TMZ) is commonly used in GBM^28^. TMZ alkylates the O^6^ positions of guanine, which leads to double strand breaks in the cancerous DNA and sensitises tumour cells to radiation^28,29^. The O^6^-methylguanine-DNA methyltransferase (MGMT) counteracts these effects via direct DNA repair^30^. Epigenetic silencing of *MGMT* via promoter methylation seems to suppress this repair mechanisms^31^. Promoter methylation of *MGMT* gene resulting in a loss of MGMT protein expression^30^. From a clinical point of view, the status of *MGMT* promoter methylation is the most important prognostic marker in GBM. *MGMT* promoter methylation is also predictive of chemotherapy responsiveness and therefore used to guide decision making with respect to chemotherapy^32^. Interestingly, Qiu and colleagues demonstrated that glioma-stem like cells derived from the naturally MGMT-negative cell line U251-MG become MGMT-positive on the protein level, which contributes to their TMZ resistance^33^. Of note, MGMT is a target gene of NF-κB signalling^34^.

In the present study, we used a CRISPR/Cas9-mediated knockout in U251-MG cells, serving as a model system for GBM, in order to study the influence of PLEKHG5 on the autophagic flux and cellular survival^35^. Specifically, we took advantage of a Cas9 nickase mutant (Cas9n) in order to induce single-strand breaks thereby minimising the possibility of off-target cleavage, which we already successfully utilised previously^35–37^. We observed changes in cell morphology and proliferation as well as a significant decrease of LAMP-1 protein in *PLEKHG5*^−/−^ cells, resulting from high-level accumulation of autolysosomes in PLEKHG5-deficient cells in comparison to the wildtype. In addition, we successfully established a constitutively active variant of RAB26 in U251-MG *PLEKHG5*^−/−^ cells, which led to a phenotypic rescue of the *PLEKHG5* knockout and revealed a PLEKHG5-specific downregulation of RhoA activity. This affection might explain the observed changes in cellular morphology of *PLEKHG5*^−/−^ cells and could be completely rescued via overexpression of RAB26. To the best of our knowledge, this is a previously not published novel report on a cross coupling, involving RAB26 and RhoA signalling. We discovered that apoptosis resulting from TNFα-mediated NF-κB activation is strongly dependent on PLEKHG5, whereas RAB26 could only partially rescue this defect in NF-κB activation. Our study also revealed a complex regulatory interplay between PLEKHG5, RAB26 and the NF-κB target gene MGMT. Expression of constitutively active RAB26 in *PLEKHG5*^−/−^ cells resulted in a robust MGMT protein expression in formerly almost MGMT-negative cells, but also increased *MGMT* promoter methylation. In summary, these data provide new insights into an important regulatory pathway in the GBM cell line U251-MG. Manipulation of and interference with this pathway may have therapeutic potential.

## Results

### Successful generation of a CRISPR/Cas9-mediated knockout of PLEKHG5 in U251-MG cells

**Figure 1:**
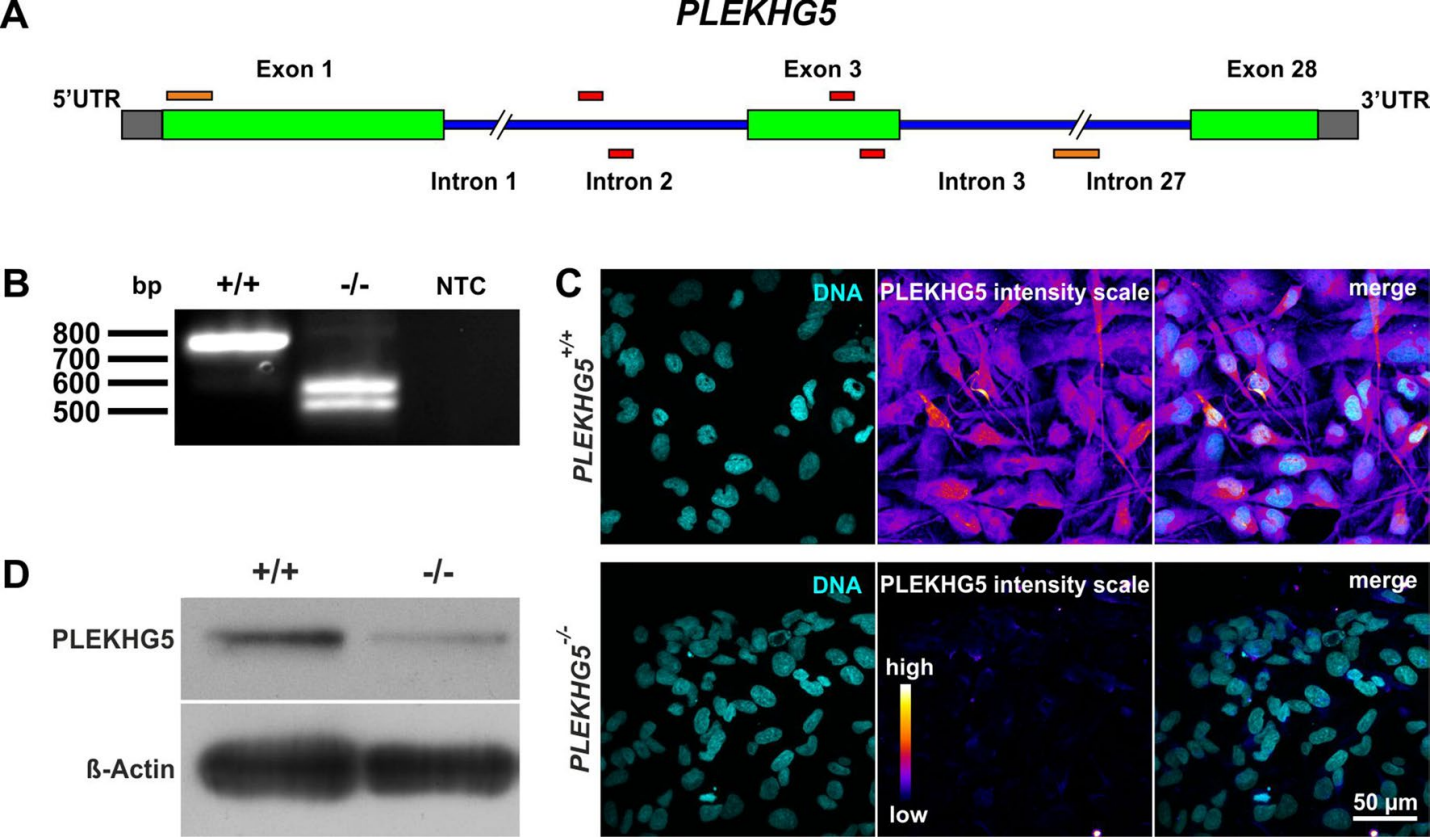
Generation and validation of the *PLEKHG5* knockout in human U251-MG cells. (**A**) Graphical representation of the *PLEKHG5* gene, including the placed sgRNAs in red and the related binding sites of the genomic primer pair in orange, spanning over the region from exon 1 to intron 4. (**B**) Genomic PCR products from clonally grown human *PLEKHG5*^+/+^ and *PLEKHG5*^−/−^ cells. *PLEKHG5* knockout shows bands around 500–600 bp in contrast to the wildtype at 750 bp. (**C**) Immunocytochemistry of *PLEKHG5*^+/+^ and *PLEKHG5*^−/−^ cells revealed the presence of PLEKHG5 in wildtype cells, which was completely lost in PLEKHG5-deficient cells. (**D**) Western blotting of whole cell lysates from *PLEKHG5*^+/+^ and *PLEKHG5*^−/−^ cells depicting the strong decrease of PLEKHG5 protein within the *PLEKHG5* knockout compared to wildtype. β-Actin served as a loading control.

We generated a genetic model in order to analyse cellular functions of PLEKHG5, in vitro. Here, we used the CRISPR/Cas9-system for the generation of a *PLEKHG5* knockout in human glioblastoma U251-MG cells. Guide RNAs including single-strand breaks were designed over the exon/intron border from intron 2 to exon 3 of the PLEKHG5 gene (Fig. 1A). Different individual clones were analysed and the homozygous clone H5.1 was further used as the *PLEKHG5* knockout. Genomic PCR of wildtype and potential knockout cells showed the successful generation of a *PLEKHG5* knockout at DNA level (Fig. 1B and Fig. S1). The nearly absence of PLEKHG5 protein in U251-MG knockout cells was validated via immunocytochemical stainings and Western blotting, depicting PLEKHG5 protein in wildtype cells. PLEKHG5 protein was almost undetectable in knockout cells after immunocytochemical staining (Fig. 1C, lower panel), in comparison to wildtype cells (upper panel). In line with the immunocytochemistry, immunoblotting of *PLEKHG5* wildtype and knockout cells depicted a drastic reduction of PLEKHG5 protein in *PLEKHG5*^−/−^ cells, compared to *PLEKHG5*^+/+^ cells (Fig. 1D and supplementary Fig. S2). In summary, a successful knockout of *PLEKHG5* could be generated in U251-MG glioblastoma cells.

### *PLEKHG5*^−/−^ U251-MG cells loose spindle shape, filopodia and have an increased population doubling time

**Figure 2:**
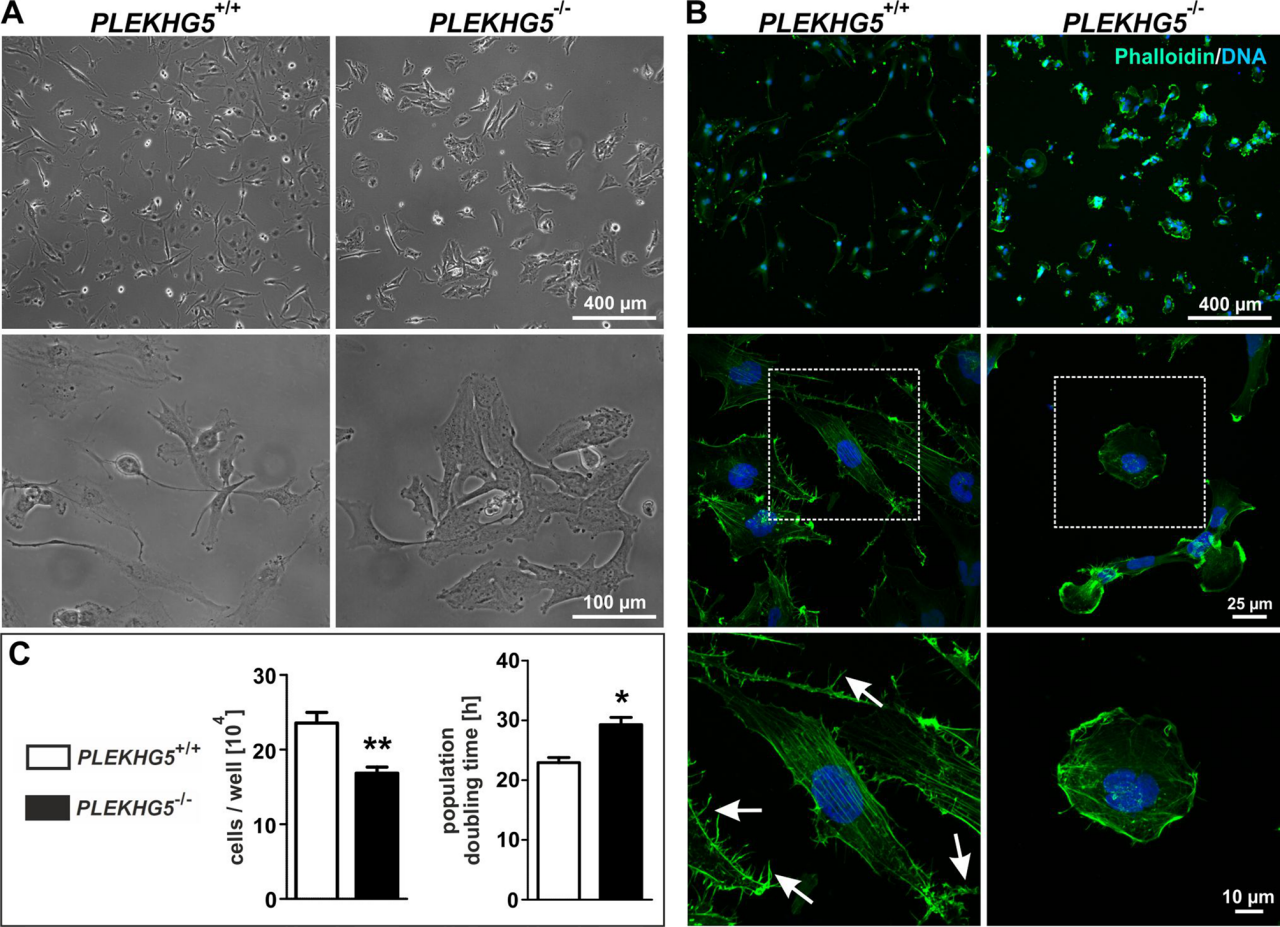
Affection of cellular structure and metabolism in *PLEKHG5* knockout in U251-MG cells. (**A**) Cultivated U251-MG *PLEKHG5*^+/+^ and *PLEKHG5*^−/−^ cells at phase contrast and various magnifications. *PLEKHG5*^−/−^ shows a bipolar/multipolar shape. (**B**) Immunocytochemical staining with phalloidin/rhodamine as cytoskeleton marker depicted the cellular affection of *PLEKHG5*^−/−^ cells, in comparison to *PLEKHG5*^+/+^ cells. Next to clustered growth, PLEKHG5-deficient cells showed a clearly decreased shape and a nearly complete loss of filopodia, which were typical in *PLEKHG5* wildtype cells and usually associated with migration (marked by arrows). (**C**) Analysis of proliferation revealed a significantly increased population doubling time of *PLEKHG5*^−/−^ (29.3 ± 1.2 h) compared to *PLEKHG5*^+/+^ cells (22.9 ± 0.9 h), which correlates with decelerated cell growth. Paired, two-tailed t tests, n = 3, **p = 0.0012 (cell growth) and *p = 0.0276 (population doubling time) were considered significant. Mean ± SEM.

We initially aimed to discover potential effects of the *PLEKHG5* knockout on the cell morphology and proliferative behaviour of U251-MG cells. Using light microscopy, we observed a change in cell shape and a more clustered growth pattern of *PLEKHG5* knockout cells, in comparison to wildtype cells (Fig. 2A). *PLEKHG5*^−/−^ cell bodies were flatted and less spindle shaped. In order to assess these effects in more detail, cultivated cells were stained with the cytoskeleton marker phalloidin/rhodamine. The bicyclic peptide phalloidin conjugated to rhodamine could be used for highly selective staining of actin filaments (also known as F-actin). The cytoskeleton of *PLEKHG5*^−/−^ cells differed considerably from its wildtype (Fig. 2B). Here, the *PLEKHG5* knockout revealed a rounded cell shape, next to decreased covered surface (Fig. 2B, middle panel). Additionally, the actin filled filopodia were nearly completely lost within the *PLEKHG5* knockout (arrow in Fig. 2B, lower panel), which are known to be involved in the migration of U251-MG and other cell lines^38^. A proliferation assay further showed a significantly prolonged population doubling time of the *PLEKHG5*^−/−^ cells, reasoned by a highly significant reduction in cell number as compared to *PLEKHG5*^+/+^ cells (Fig. 2C).

### Accumulation of autolysosomes in PLEKHG5-deficient cells is associated with a strong decrease in LAMP-1 protein

**Figure 3:**
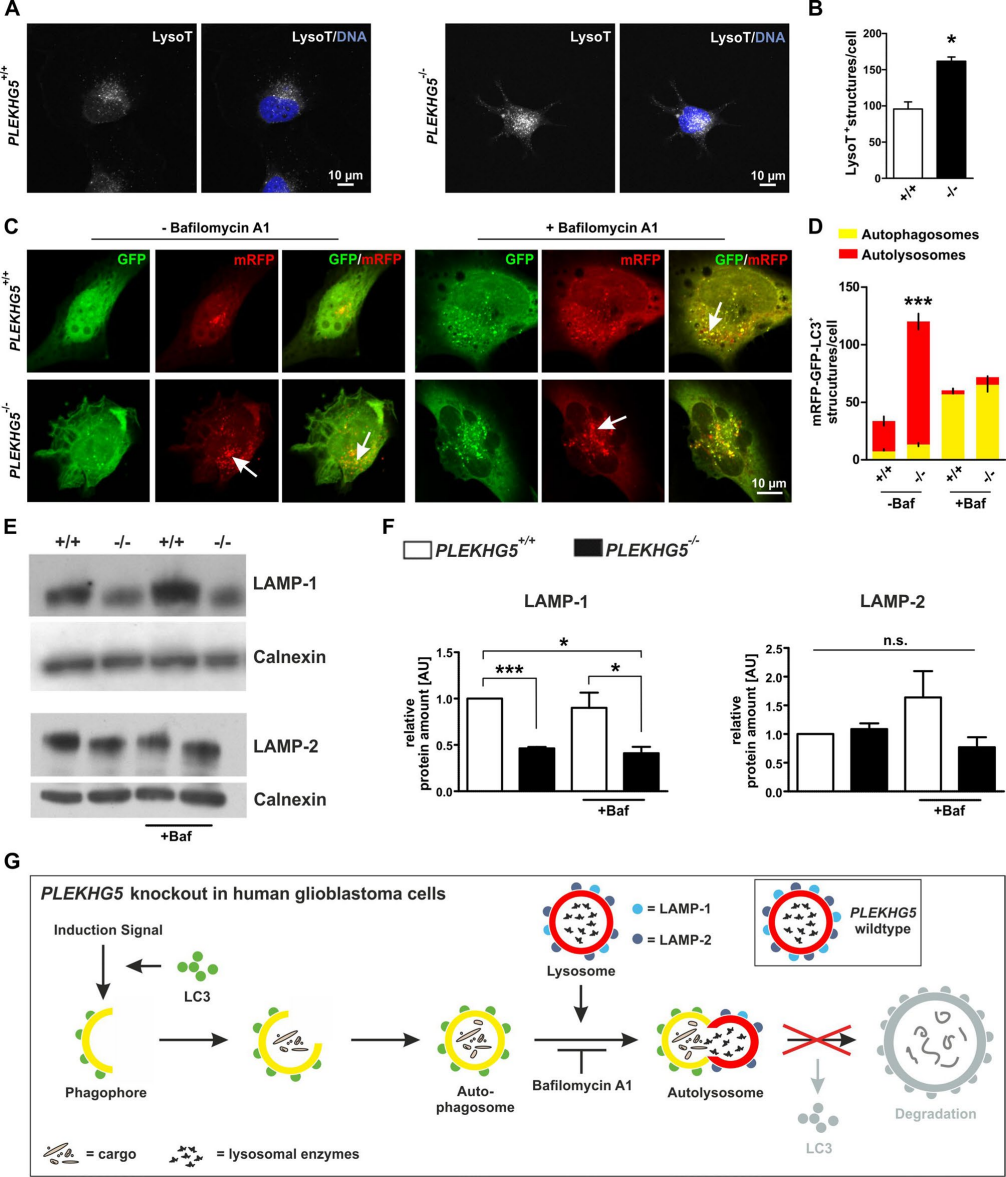
Accumulation of autolysosomes and specific decrease of LAMP-1 in PLEKHG5-deficient cells. (**A**) and (**B**) Staining of acidic organelles using LysoTracker deep red indicates accumulation of autophagy-involved structures in *PLEKHG5*^−/−^ cells. Unpaired, two-tailed t test, five cells per experiment and genotype were analysed, n = 3, **p = 0.0125 was considered significant. (**C**) and (**D**) Immunocytochemistry of PLEKHG5-deficient and wildtype cells, after transduction of the mRFP-GFP-LC3^+^ reporter construct, followed by a partially treatment of Bafilomycin A1. Quantification of immunocytochemical stainings by measuring the autophagic associated structures per cell (exemplary marked by arrows) was divided in the number of autophagosomes (mRFP^+^-GFP^+^-LC3) and autolysosomes (mRFP^+^-GFP^−^-LC3). Autophagosomes accumulated as expected by treatment with Bafilomycin A1, which blocks the fusion of autophagosomes and lysosomes but remain unchanged in the different conditions between mutant- and wildtype cells. In contrast, the autolysosomal amount (red segment) was significantly enhanced only in *PLEKHG5*^−/−^ cells at basal conditions (106.9 ± 11.9) when compared to wildtype cells (26.9 ± 6.9). Two-way ANOVA with Bonferroni post-test, five cells per experiment and genotype, n = 3, ***p < 0.0001 was considered significant. (**E**) Western blot analysis of Bafilomycin A1 treated and untreated U251-MG wildtype/*PLEKHG5*^−/−^ cell lysates. Immunodetection of LAMP-1 revealed an irregular protein amount, which differed between both phenotypes. *PLEKHG5* wildtype cells showed in every condition a stronger signal for LAMP-1 protein, whereas these signals decreased obviously in lysates from PLEKHG5-deficient cells. Calnexin served as an ER marker for normalisation (as published by Lüningschrör et al*.*^9^). (**F**) Quantification of LAMP-1 protein amounts normalised to amounts of Calnexin, depicting the strongly significant decrease of LAMP-1 in PLEKHG5-deficient cells (knockout 0.46 ± 0.02 vs. wildtype 1.0 ± 0.00). Paired, two-tailed t test, n = 3, ***p = 0.0008, *p = 0.0405 (comparison after Bafilomycin A1 treatment) and * *p* = 0.0137 (untreated wildtype vs. treated knockout) were considered significant. Mean ± SEM. (**G**) Schematic representation of autophagy in PLEKHG5-deficient U251-MG cells (modified from Jing et al.^21^). Genomic deletion of *PLEKHG5* leads to an impairment of lysosomal degradation, reasoned by significantly downregulation of LAMP-1 within an accumulation of autolysosomes.

In addition to changes in morphology and proliferative behaviour, potential alterations of autophagic activity were assessed in the *PLEKHG5* knockout. Cancer cells commonly show enhanced autophagic activity^39^ and there is a correlation between autophagy and increased cell survival in GBM^40^. We stained acidic organelles with LysoTracker deep red to visualise autophagic processes within *PLEKHG5* wildtype and knockout cells. *PLEKHG5* knockout cells revealed a significantly increased amount of LysoTracker deep red-stained acidic organelles compared to the wildtype (Fig. 3A, B). To analyse these effects in more detail, the mRFP-GFP-LC3^+^ reporter was stably introduced into U251-MG *PLEKHG5*^+/+^ and *PLEKHG5*^−/−^ cells, which functions as a fusion-protein of mRFP/GFP and a LC3^+^ tracing system^41^. LC3 is commonly used to monitor autophagy. In particular, the LC3-II is an autophagosome marker^42^. While the formation of autophagosomes can therefore be measured as an accumulation of the GFP-positive LC3-II activity, the formation of autolysosomes leads to a pH decrease and the loss the autophagosome associated GFP signal. The mRFP signal is less sensitive to pH changes and can be used as a marker for autolysosomes. Quantification of the number of mRFP^+^-GFP^+^-autophagosomes and mRFP^+^-GFP^−^ autolysosomes by counting the fluorescent structures in each single cell showed a strongly significant accumulation of autolysosomes in PLEKHG5-deficient compared to wildtype cells (Fig. 3C, D). Next, *PLEKHG5* wildtype and knockout cells were treated with Bafilomycin A1 in order to block the fusion of autophagosomes and lysosomes (see Fig. 3G for the site of action of Bafilomycin A1). An increase of endogenous LC3-II-positive autophagosomes was seen in both *PLEKHG5*^+/+^ and *PLEKHG5*^−/−^ cells after treatment with Bafilomycin A1, but no differences were seen between Bafilomycin A1-treated *PLEKHG5* wildtype and knockout cells. We conclude that PLEKHG5 interferes with autophagic flux before autolysosomes are formed (Fig. 3D). Next, the predominant lysosomal surface markers LAMP-1 and -2 were investigated in more detail. Together, LAMP-1 and -2 may contribute up to 50% of the proteins of the lysosomal membrane^43^.

We analysed the amounts of LAMP-1 and -2 protein in U251-MG *PLEKHG5* knockout and wildtype cells before and after Bafilomycin A1 treatment with Western blots. *PLEKHG5* knockout cells contained strongly and significantly reduced amounts of LAMP-1 protein, in comparison to wildtype cells (Fig. 3E, F and Fig. S3). In contrast, no statistically significant differences in LAMP-2 protein expression were observed between *PLEKHG5*^−/−^ and *PLEKHG5*^+/+^ cells.

### Phenotypic rescue of U251-MG *PLEKHG5*^−/−^ cells with RAB26

**Figure 4:**
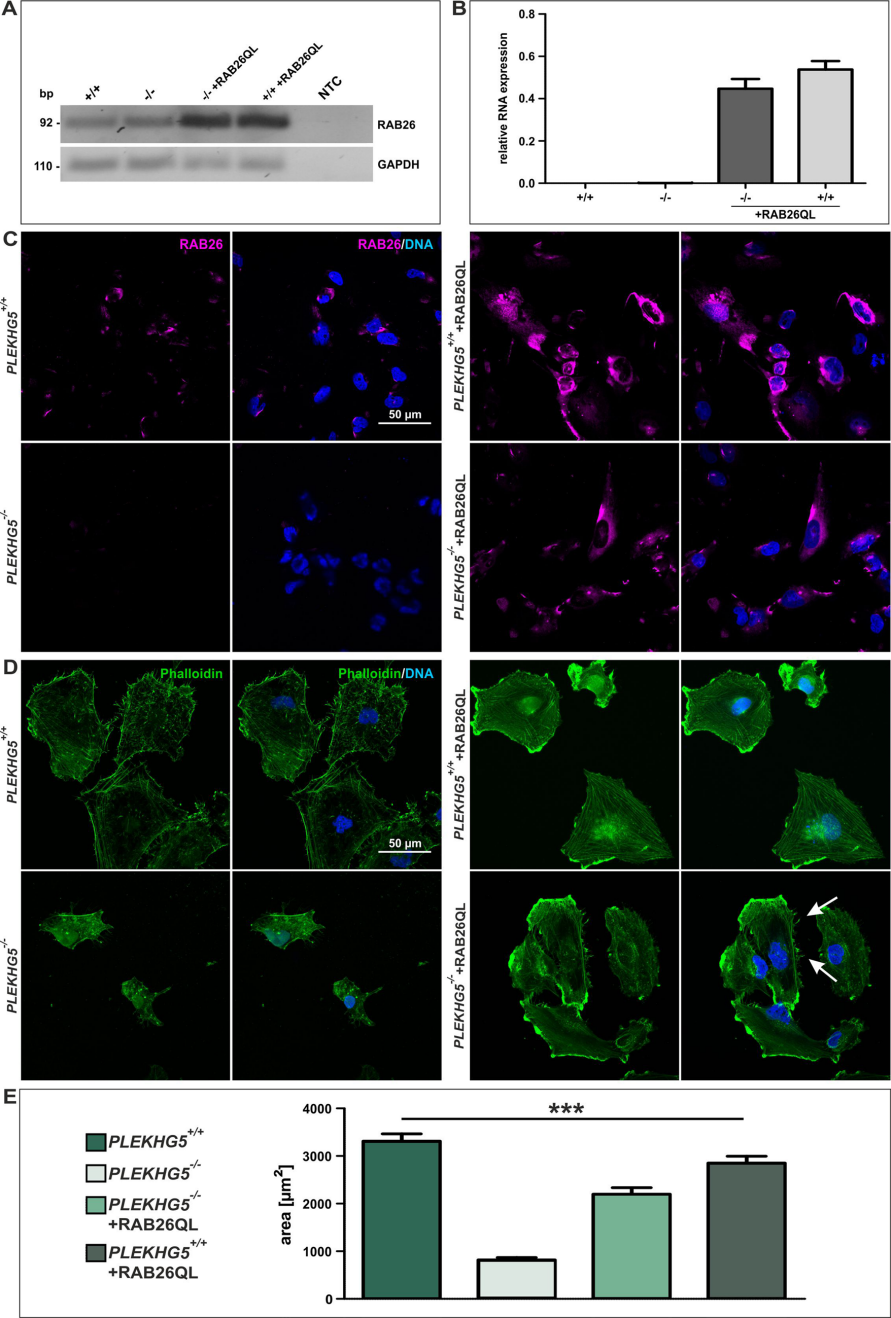
Lentivirus-mediated overexpression of RAB26 resulted in phenotypic rescue of U251-MG *PLEKHG5*^−/−^ cells. (**A**) Initial detection of different RAB26 expressions in partial lentiviral transduced U251-MG cells, indicated a successful RAB26 overexpression in *PLEKHG5*^−/−^ + RAB26QL (rescue) as well as in *PLEKHG5*^+/+^ + RAB26QL cells, at post-transcriptional level. (**B**) Quantitative mRNA analysis via qRT-PCR verified the high-level overexpression of *RAB26* gene in transduced U251-MG cells with RAB26QL constructs (0.54 ± 0.04 and 0.45 ± 0.05), compared to the expression of *PLEKHG5* wildtype and knockout cells (both ≤ 1.2 × 10^−3^ ± 0.0001) without constitutively active RAB26QL. (**C**) Immunocytochemistry of partial lentiviral transduced U251-MG cells with the RAB26QL construct showed also highly RAB26 expressions in *PLEKHG5*^−/−^ and *PLEKHG5*^+/+^ cells with constitutive active RAB26 on protein level. (**D**) Staining with phalloidin/rhodamine depicted morphologic changes in wildtype and knockout cells, due to transduced U251-MG cells, whereas the loss of filopodia and the affection of the cell size from *PLEKHG5* knockout cells were restored (filopodia linked with arrows). (**E**) Effects at cell size could be identified as strongly significant during area analysing. Mann–Whitney U tests, n = 3 (min. 20 cells for each biological replicate), ***p < 0.0001 for wildtype: 3313 ± 153 µm^2^ vs. knockout: 817 ± 51 µm^2^, knockout: 817 ± 51 µm^2^ vs. *PLEKHG5*^−/−^ + RAB26QL: 2199 ± 136 µm^2^ and *PLEKHG5*^−/−^ + RAB26QL: 2199 ± 136 µm^2^ vs. *PLEKHG5*^+/+^ + RAB26QL: 2851 ± 143 µm^2^ were considered significant. Mean ± SEM.

We previously discovered that RAB26 functions as a G-protein for PLEKHG5^9^. Hence, we analysed whether the same pathway might also be active in U251-MG cells. To this end, *PLEKHG5*^+/+^ and *PLEKHG*5^−/−^ U251-MG cells were transduced with cFUGW-EGFP-RAB26QL, a mutant (QL mutation) and constitutively active variant of the Ras-related protein RAB26 linked to EGFP, in order to allow for fluorescence tracing. As a control, lentivirus transduced U251-MG cells depicted the presence of EGFP-positive cells after transduction (Fig. S4). Overexpression of RAB26 in cells transduced with cFUGW-EGFP-RAB26QL was confirmed with quantitative RT-PCR analysis (Fig. 4A, B and Fig. S1) using GAPDH (Fig. 4A, B) and RpL0 (Fig. 4B) as housekeeping genes as well as via immunocytochemical stainings of RAB26 protein. Immunocytochemistry of RAB26 showed high amounts of RAB26 protein in transduced *PLEKHG5* wildtype and knockout cells, while only moderate expression levels were found in non-transduced *PLEKHG5*^+/+^ cells and no RAB26 protein in non-transduced *PLEKHG5*^−/−^ cells (Fig. 4C). Cytoskeleton analysis with fluorescence imaging after phalloidin/rhodamine staining showed a significantly increased average cell surface by measuring of the stained cellular areas and filopodia, in contrast to *PLEKHG5*^−/−^ cells following transduction with RAB26QL (Fig. 4D, E). Additionally, *PLEKHG5*^+/+^ + RAB26QL cells depicted an intermediate of measured cellular area between transduced *PLEKHG5* knockout and *PLEKHG5* wildtype cells.

**Figure 5:**
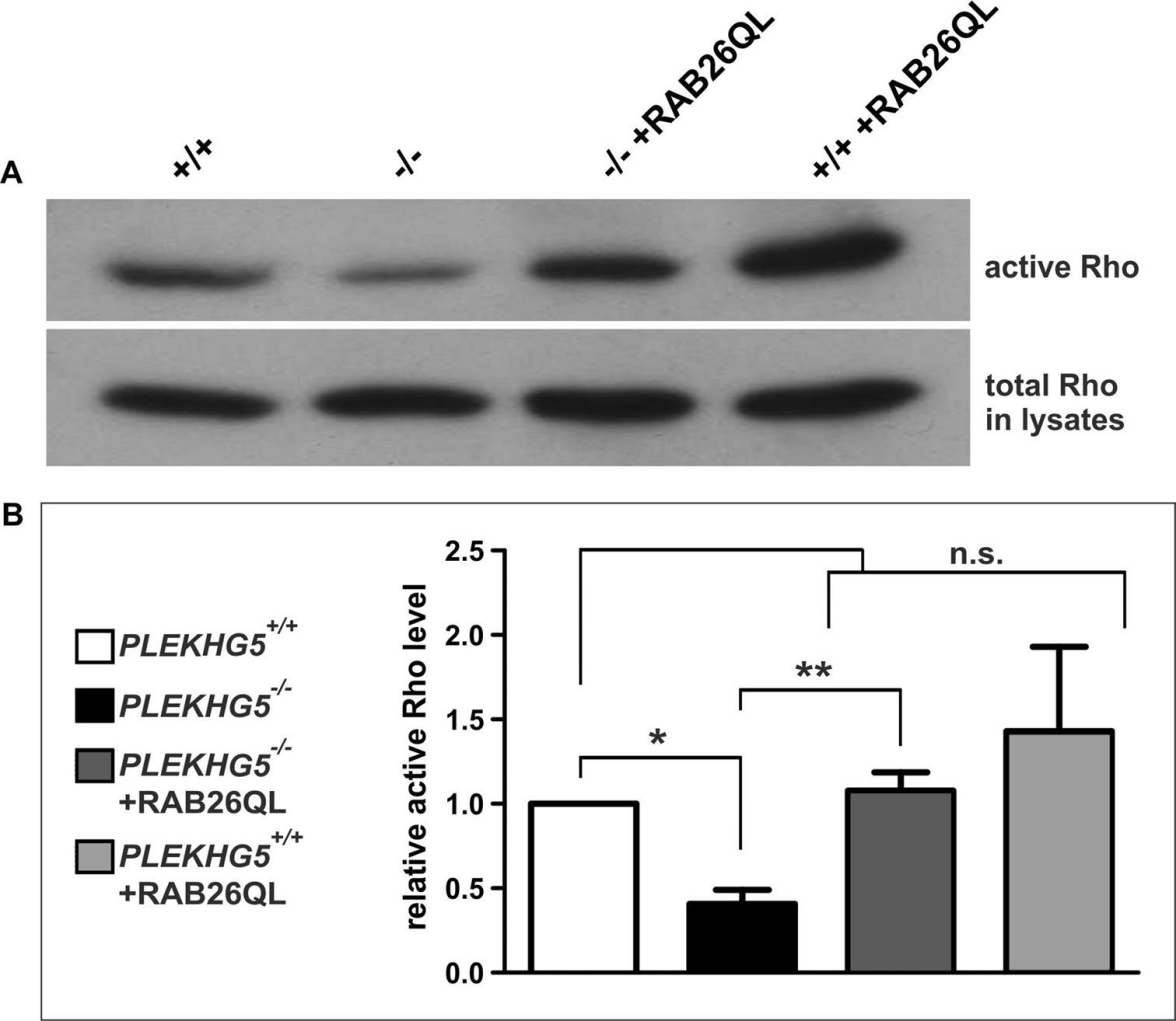
Rescue of *PLEKHG5* knockout via RAB26 restored the depletion of RhoA activity in U251-MG. (**A**) Immunoblotting of fractionated U251-MG cell lysates depicted amounts of active RBD-bound RhoA and the quantity of the total RhoA protein after GST-RBD pulldown assay. (**B**) Quantification of Western blots showing the determined relative active RhoA levels normalised to the total RhoA protein amount in whole cell lysates. Significant reduction of active RhoA in PLEKHG5-deficient cells compared to U251-MG wildtype could be restored after phenotypic rescue in *PLEKHG5*^−/−^ + RAB26QL cells. No significantly changes could be examined between *PLEKHG5*^+/+^ and *PLEKHG5*^−/−^ + RAB26QL or *PLEKHG5*^+/+^ + RAB26QL cells. Statistical analysis were performed via paired, two-tailed t test, n = 3, **p = 0.005 and *p = 0.020 were considered significant. Mean ± SEM.

To further analyse involved signal transduction pathways in the changes within the cytoskeleton, we performed a Rho GST-RBD pulldown assay. This might allow assessing an involvement of RhoA. Thus, we analysed, if possible changes in the amounts of active RhoA within the *PLEKHG5* knockout could be rescued with an overexpression of RAB26. Therefore, U251-MG cells were cultured, harvested and whole protein lysates were produced for the following GST-RBD pulldown assay. Probes were analysed by Western blotting (Fig. 5). As an indication, *PLEKHG5*^−/−^ cells seemed to have less amounts of active RhoA protein, in contrast to *PLEKHG5*^+/+^ cells or U251-MG cells, which were transduced with RAB26QL constructs (Fig. 5A and Fig. S2). Statistical analysis approved this suspicion, whereas the specific RhoA activity of *PLEKHG5* wildtype cells were significantly downregulated in *PLEKHG5* knockout cells but could be completely rescued in transduced *PLEKHG5*^−/−^ cells, overexpressing RAB26 (Fig. 5B). Also, *PLEKHG5*^+/+^ + RAB26QL cells depicted similar relative active RhoA levels as the *PLEKHG5* wildtype and the *PLEKHG5* rescue. In summary, our findings clearly demonstrate that overexpression of RAB26, a G-protein for PLEKHG5, resulted in a phenotypic rescue of U251-MG *PLEKHG5*^−/−^ cells, which restored the affection of the cytoskeleton, resulted by a downregulation of the RhoA activity.

### PLEKHG5 expression in U251-MG cells leads to NF-κB activation and is involved in the regulation of apoptosis

**Figure 6:**
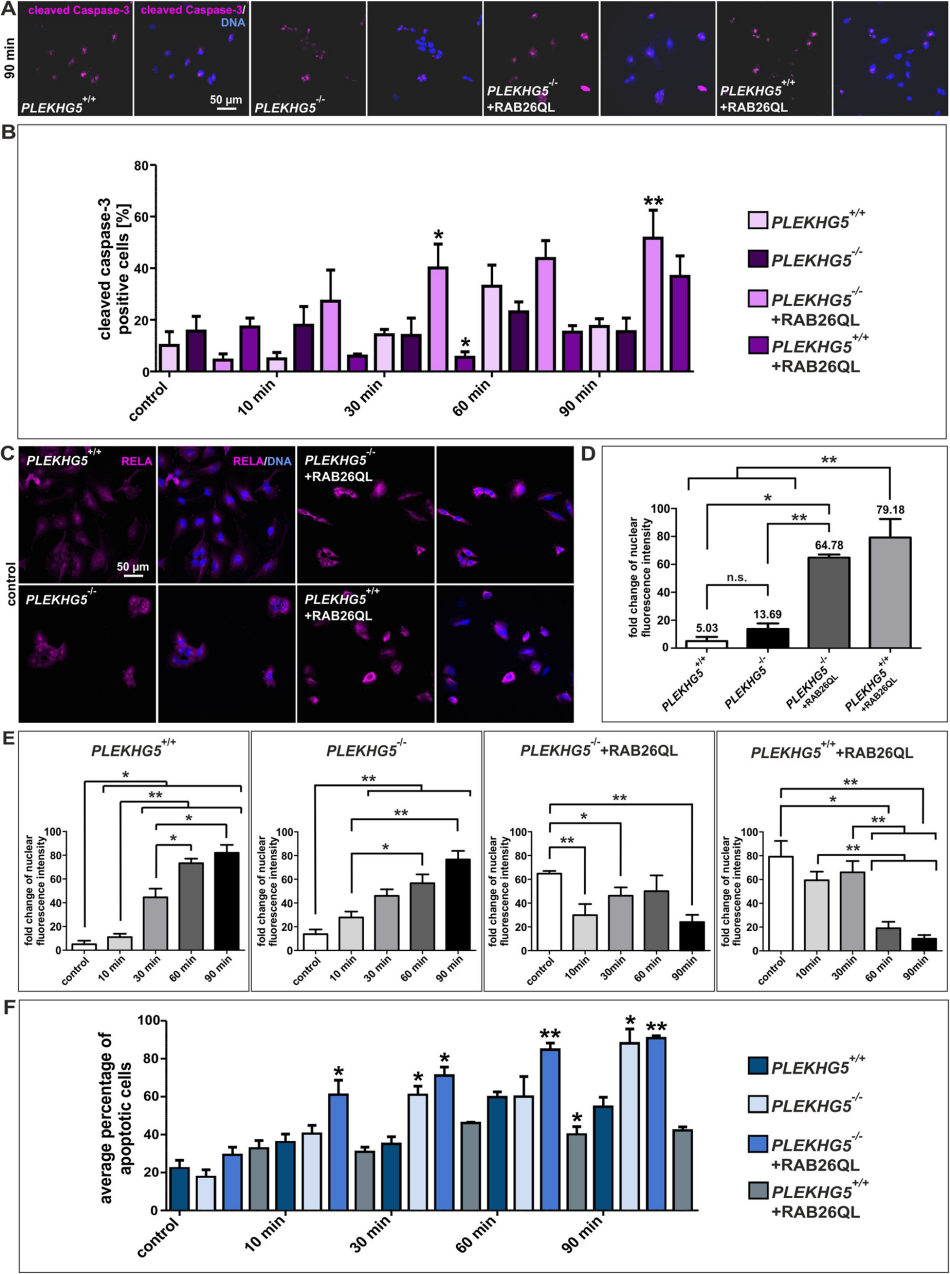
TNFα induced apoptosis in U251-MG, after PLEKHG5-mediated NF-κB activation. (**A**) and (**B**) Immunocytochemistry depicting cleaved Caspase-3 after 90 min of TNFα stimulation (**A**). Indicated discrepancies between *PLEKHG5* wildtype, knockout and transduced U251-MG cells were examined at different periods of TNFα stimulation, compared to untreated controls (exemplary pictures in Fig. S5). Resulting effects were determined via the percentage of cleaved Caspase-3 positive cells, compared to the total amount of cells for the respective condition and cell line (**B**). Statistics were performed via comparison with *PLEKHG5* wildtype. No clear trend could be visualised, except in *PLEKHG5*^−/−^ + RAB26QL cells after 30/90 min of stimulation. Cells depicted significantly higher amounts of protein, indicating differences in the apoptosis compared to *PLEKHG5* wildtype. (**C**) Immunocytochemistry of RELA indicated differences of the NF-κB transcription factor activity in U251-MG cells. Whereas *PLEKHG5* wildtype and knockout seemed to depict lower levels of nuclear RELA, compared to U251-MG cells with RAB26 overexpression. (**D**)–(**F**) Comparison of untreated cells (**D**) and effects of TNFα stimulation (exemplary pictures in Fig. S6) were measured via fold change of nuclear fluorescence intensity (**D**, **E**) and with the average percentage of apoptotic cells (**F**) grouped at the different time points, compared to *PLEKHG5* wildtype cells. Nuclear fluorescence intensity were analysed via measuring RELA expressions in the nuclei. Apoptotic rates were determined by the state and size of nuclei, based at the examination of DAPI staining (**F**). Whereas *PLEKHG5* wildtype and knockout depicted clearly trends from cytoplasmic up to nuclear expressions of RELA, *PLEKHG5*^−/−^ + RAB26QL and *PLEKHG5*^+/+^ + RAB26QL cells showed a nearly perinuclear signal in all periods after stimulation with TNFα. Analysis of TNFα-induced apoptosis showed that the fitness of U251-MG is relating to the specific PLEKHG5 activation. In addition, U251-MG cells with overexpression of RAB26 displayed enhanced base levels at the fold change of nuclear fluorescence intensity as well as a significantly faster response to TNFα as *PLEKHG5*^+/+^and *PLEKHG5*^−/−^ cells. All statistical analysis were performed with Mann–Whitney U tests (min. 47 cells for each condition and cell line), **p ≤ 0.01 and *p ≤ 0.05 were considered significant. Mean ± SEM.

**Figure 7:**
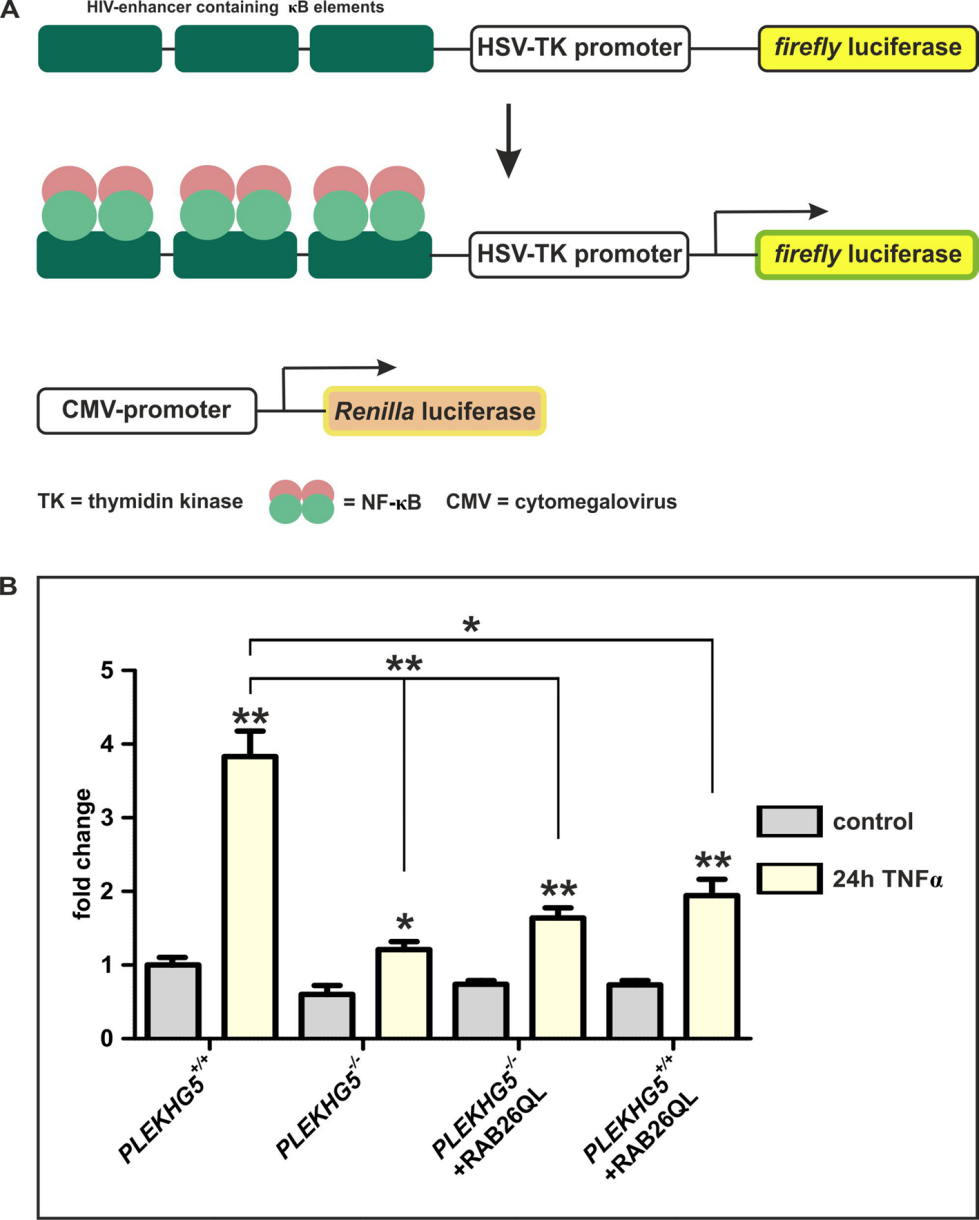
Dual luciferase reporter system revealed strong reduction of NF-κB activity in PLEKHG5-deficient cells after TNFα stimulation. (**A**) Schematic representation of the used NF-κB-reporter system containing TK(NF-κB)_6_LUC vector for detecting NF-κB-activity via *firefly* luciferase and the constitutively expressing *Renilla* luciferase vector pRLcmv for normalisation (scheme modified from Greiner et al.^58^). (**B**) Detection of the NF-κB reporter after 24 h of TNFα treatment showed significantly reduced levels of luciferase activity in PLEKHG5-deficient cells compared to *PLEKHG5* wildtype cells. Transduced U251-MG cells also depicted a reduction in the NF-κB activity after TNFα stimulation compared to *PLEKHG5* wildtype cells, indicating a more PLEKHG5-dependent regulation as correlated to RAB26. In each cell line, the specific NF-κB activation after TNFα treatment was determined as statistically significant, in comparison to the respective control. Statistical analysis were performed via unpaired, two-tailed t tests (three analysis for each condition and cell line), **p ≤ 0.01 and *p ≤ 0.05 were considered significant. Mean ± SEM.

PLEKHG5 is an upstream activator of the transcription factor NF-κB^10^ and interference with autophagy can lead to NF-κB activation^44^. Together, this prompted us to analyse NF-κB activity in PLEKHG5-deficient and wildtype cells with and without the NF-κB activating stimulus TNFα. RELA (p65) plays a central role in the classical pathway of NF-κB activation and nuclear RELA expression was therefore used as a measure of NF-κB activity. Since TNFα is a potent inducer of apoptosis, we also studied the impact of RAB26 expression on apoptotic cell death after TNFα stimulation (Fig. 6). Therefore, we firstly treated U251-MG cell lines with TNFα and stained the cells afterwards immunocytochemically with cleaved Caspase-3, which is a commonly used method for analysing the cellular apoptosis (Fig. 6A and Fig. S5). Surprisingly, induced apoptosis was increased predominantly in *PLEKHG5* rescue cells via RAB26QL as compared to *PLEKHG5*^+/+^ cells with the same construct (Fig. 6B). This unexpected effect, whereas no significant trend could be observed within the analysed *PLEKHG5* wildtype, knockout and wildtype cells overexpressing RAB26, might be reasoned by the given fact that the activation of cleaved Caspase-3 can take up to 72 h in U251-MG cells^45^. Thus, we analysed in addition apoptosis in RELA positive cells (Fig. 6C–F and Fig. S6). Treatment of *PLEKHG5*^+/+^ as well as *PLEKHG5*^−/−^ cells with TNFα resulted in a > 70-fold increase of nuclear RELA (Fig. 6E). In contrast, U251-MG cells overexpressing RAB26 were found to express much higher base levels of RELA protein, while exposure to TNFα resulted in relative suppression of RELA expression (Fig. 6D, E). As a result of a not strictly trend after cleaved Caspase-3 stainings, the cellular fitness after TNFα stimulation was also examined by analysing the average percentage of apoptotic cells via DAPI staining (Fig. 6F and Fig. S6). Here, U251-MG cells with a nuclear condensation were designated as clearly apoptotic, whereby it is commonly reported by Koopman and colleagues that the detection of apoptosis by using DAPI is directly correlated with an Annexin V positivity of analysed cells^46^. After 30 min and 90 min of treatment with TNFα, the fitness of U251-MG differed significantly between *PLEKHG5* knockout, *PLEKHG5* rescue and *PLEKHG5* wildtype cells. No statistically significant induction of apoptotic cells could be seen in *PLEKHG5*^+/+^ + RAB26QL cells after TNFα treatment. The lowest apoptotic rates were seen in *PLEKHG5*^+/+^ and *PLEKHG5*^+/+^ + RAB26QL cells, while a low (*PLEKHG5*^−/−^) activity of the pathway increased TNFα-induced apoptotic death. Thus, the absence of PLEKHG5 seemed to increase NF-κB activation in unstimulated U251-MG cells. To address this point in detail, we transfected U251-MG cell lines with a NF-κB reporter and control plasmids, to analyse the TNFα-specific activation of the NF-κB pathway (Fig. 7). Specific luciferase activities were analysed and measured in comparison to the respective untreated control (Fig. 7A, B). As a result, we could not observe any statistically significant changes between the untreated controls of the different U251-MG cell lines. However, the specific NF-κB activation after TNFα stimulation in *PLEKHG5* knockout cells was significantly reduced as compared to *PLEKHG5* wildtype cells. This effect seemed to be only slightly rescued with the overexpression of RAB26 in *PLEKHG5*^−/−^ + RAB26QL cells and even *PLEKHG5*^+/+^ + RAB26QL cells displayed an intermediate luciferase activity between *PLEKHG5* wildtype and *PLEKHG5* rescue cells. Taken together, our findings suggest that TNFα-mediated NF-κB activation is strongly dependent on PLEKHG5. RAB26 could only partially rescue the defect in TNFα-mediated NF-κB activation.

### RAB26 overexpression increases expression of the NF-κB target gene MGMT

**Figure 8:**
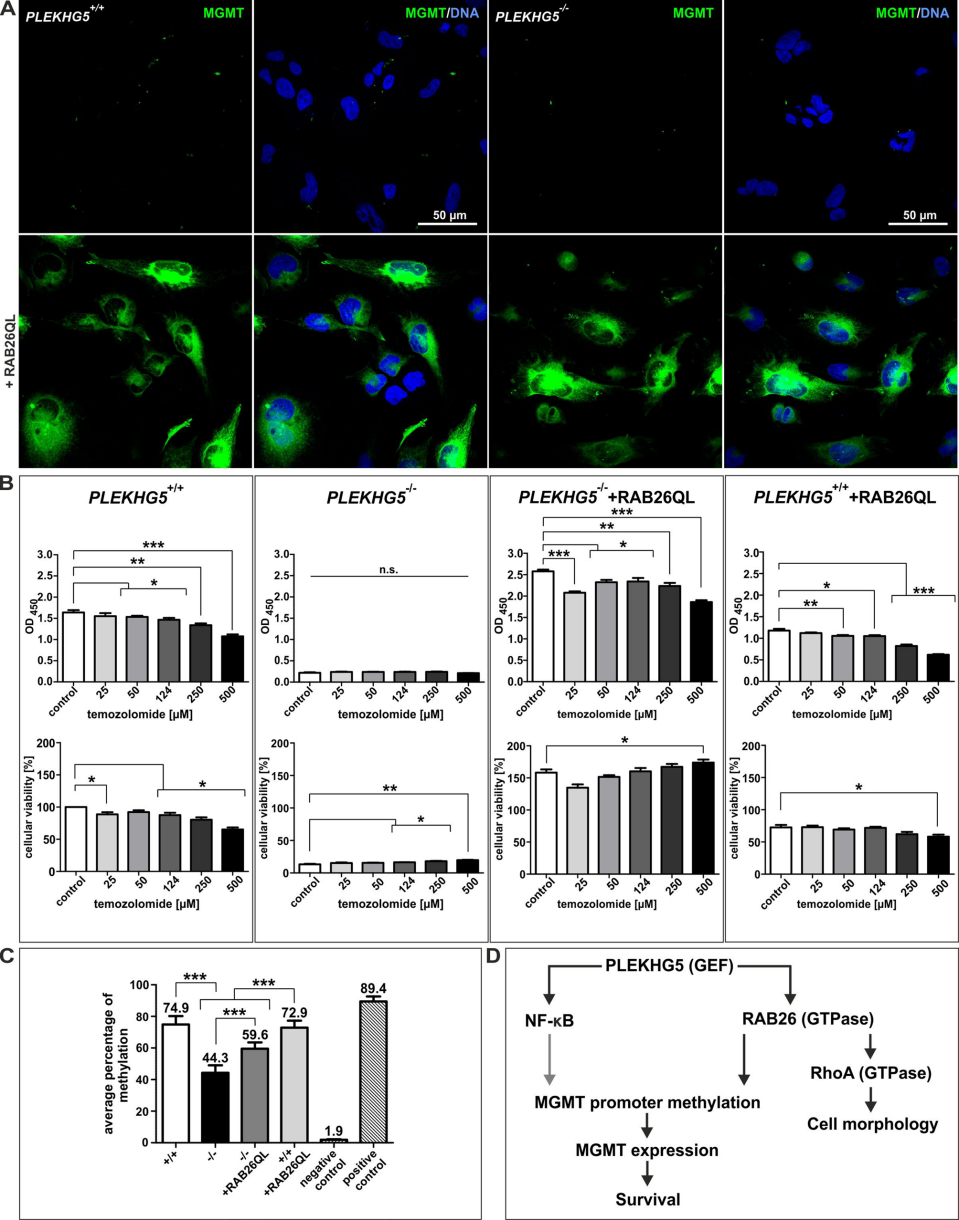
PLEKHG5 regulates NF-κB and MGMT in U251-MG. (**A**) Immunocytochemistry showed strong accumulations on MGMT expression after transduction with RAB26QL. Overexpression of RAB26 seemed to correlate with the amounts of MGMT protein, whereas *PLEKHG5* rescue cells represented high levels of MGMT. (**B**) Survival analysis were performed by using Orangu Cell Proliferation Assay via measurement of photometric absorbance at 450 nm (upper row) and via determination of resulting cellular viability (lower row), after treatment with TMZ. Viability percentages of *PLEKHG5* knockout and transduced U251-MG cells were examined compared to wildtype viabilities for each condition. *PLEKHG5*^+/+^ cells showed significant decreases of OD’s/viabilities from control to treated cells. PLEKHG5-deficient cells depicted in all conditions very low levels of metabolic activity/cellular viabilities, compared to *PLEKHG5* wildtype. Overexpression of RAB26 in *PLEKHG5*^−/−^ + RAB26QL cells recovered the sensitivity to TMZ, similarly to *PLEKHG5* wildtype and rescued the cellular viability completely as compared to *PLEKHG5*^+/+^ cells. *PLEKHG5*^+/+^ + RAB26QL cells showed slightly lower levels of cellular viabilities but a similar trend as *PLEKHG5* wildtype. Statistical analysis were performed with Mann–Whitney U tests (eight analysis for each condition and cell line), ***p ≤ 0.001, **p ≤ 0.01 and *p ≤ 0.05 were considered significant. Mean ± SEM. (**C**) *MGMT* promoter methylation status of U251-MG and technical controls were analysed via bisulfide sequencing, depicting an average of 74.9 ± 5.3% methylated CpG-islands in *PLEKHG5* wildtype, compared to *PLEKHG5* knockout with 44.3 ± 4.8% of promoter methylation. Constitutive activity of RAB26QL in U251-MG cells displayed an intermediate phenotype with a promoter methylation average of 59.6 ± 4.0% (*PLEKHG5* rescue) and 72.9 ± 4.4% (*PLEKHG5*^+/+^ + RAB26QL). No statistical difference was examined between *PLEKHG5*^+/+^ and *PLEKHG5*^+/+^ + RAB26QL. One-way ANOVA with Bonferroni's multiple comparison test, ***p ≤ 0.001 (grouped for each cell line and CpG-islands) was considered significant. Mean ± SEM. (**D**) Schematic representation of the analysed relation from PLEKHG5 to its downstream targets (black arrows). Next to regulating MGMT expression and cell morphology, PLEKHG5 was revealed to modulate cellular viability, which is directly associated with U251-MG cell survival. Grey arrow represents a previously published relation, which was not addressed in this study.

Next, we examined the relation between RAB26 expression and expression of the NF-κB target gene MGMT^34^ (Fig. 8, see Fig. 8D for a scheme). Of note, increased MGMT expressions have been associated with TMZ resistance in glioma stem-like cells as well as in glioblastoma xenograft models^34,47^. Similarly, hypomethylation of the *MGMT* promoter presumably resulting in increased MGMT expression, has been correlated with TMZ chemoresistance and worse survival in glioblastoma patients^2^. Here, U251-MG cells were immunocytochemically stained for their MGMT expression (Fig. 8A). Whereas MGMT expression was barely detectable in *PLEKHG5*^+/+^ and *PLEKHG5*^−/−^ cells, U251-MG cells with RAB26 overexpression showed high-level accumulation of MGMT perinuclear and within the cytoplasm. As a verification of the used antibody, we also stained HeLa cells via immunocytochemistry, which were commonly known to express MGMT on protein level^48^ (Fig. S7). As it was expected, cultured HeLa cells depicted MGMT expression with a nuclear/perinuclear and cytoplasmic localisation, nearly like U251-MG cells with RAB26 overexpression. These data suggest a complex signalling pathway from PLEKHG5 through NF-κB activation on one side (see Figs. 6D, E and 7) and a signal transduction from RAB26 to a high protein expression of the NF-κB target gene MGMT, on the other side (Fig. 8A). As detailed above (2.2. and 2.4) the PLEKHG5/RAB26 pathway is involved in the regulation of U251-MG cell proliferation, cellular morphology and PLEKGH5-deficient cells have much higher population doubling times. We therefore investigated how the activity of the PLEKHG5/RAB26 pathway might influence the cellular viability (Fig. 8B). As expected, PLEKHG5-deficient cells showed only very low levels of cellular viability when compared to wildtype U251-MG cells using to assess survival. Overexpression of RAB26 in *PLEKHG5*^−/−^ + RAB26QL cells restored the proliferative phenotype of U251-MG cells compared to *PLEKHG5*^+/+^ cells but could not be increased further in *PLEKHG5*^+/+^ + RAB26QL cells. Additionally, the proliferative activity could not be enhanced in comparison to the *PLEKHG5* wildtype. The slight decrease in cellular viability of *PLEKHG5*^+/+^ + RAB26QL cells in comparison wildtype cells, could therefore be explained with an overexpression of RAB26 (while regular expression of PLEKHG5) but the involved pathways are not yet known. Exposure to the chemotherapeutic agent TMZ had only moderate effects on U251-MG cell survival. Nevertheless, some anti-proliferative effects were seen in *PLEKHG5* wildtype and in transduced U251-MG cells, but not in *PLEKHG5*^−/−^ cells. This may be very cautiously interpreted as evidence that TMZ chemosensitivity may require some PLEKHG5/RAB26 activation but not in a direct correlation to their specific MGMT expression in U251-MG cells. Since TMZ resistance in GBM correlates with *MGMT* promoter hypomethylation, we determined the *MGMT* promoter methylation status in U251-MG cell lines using a standard diagnostic assay (Fig. 8C and Fig. S4). Interestingly, *PLEKHG5*^+/+^ and transduced U251-MG cells showed very high degrees of *MGMT* promoter methylation (wildtype: 74.9% ± 5.3%, rescue: 59.6% ± 4.0% and wildtype + RAB26QL: 72.9% ± 4.4%), while the *PLEKHG5*^−/−^ cells displayed the lowest promoter methylation (44.3% ± 4.8%). This could be carefully interpreted, as a RAB26 expression related direction of the *MGMT* promoter methylation but seemed to be an extremely complex interaction, which needs further investigation. In the context to the amount of methylation, it was published that a clinical glioblastoma with a similar degree (i.e. 40–80%) of *MGMT* promoter methylation would be diagnosed as *MGMT* promoter hypermethylated^49^. In conclusion, these findings revealed a highly complex interplay between PLEKHG5/RAB26, MGMT expression as well as promoter methylation and the specific cellular viability or the sensitivity to TMZ.

## Discussion

PLEKHG5 has been investigated as a novel prognostic biomarker for glioblastomas^7^. In order to study the role of PLEKHG5 in more detail, we developed a model for PLEKHG5 function in the glioblastoma cell line U251-MG. Using the CRISPR/Cas9 system, we generated *PLEKHG5*^−/−^ cells devoid of any PLEKHG5 protein. This resulted in abnormal cell morphology and a loss of filopodia, which are important for cell migration. Interestingly, Qian and co-workers reported low cellular migration after knockdown of *PLEKHG5* in U251-MG^7^. In addition, *PLEKHG5*^−/−^ cells were characterised by a substantial reduction of cell viability, proliferation and cell growth.

PLEKHG5 has been identified as a specific GEF for the Ras superfamily member and GTPase RAB26^9^. Hence, we investigated if at least some of the effects of PLEKHG5 might in fact be mediated by RAB26. To this end, we established a PLEKHG5-deficient U251-MG cell system constitutively overexpressing RAB26 (U251-MG *PLEKHG5*^−/−^ + RAB26QL). Indeed, constitutive RAB26 expression rescued many of the effects of PLEKHG5 deficiency on cellular morphology, proliferative properties as well as in the regulation of RhoA activity. Our study provides some novel mechanistic insights with respect to the role of PLEKHG5/RAB26 signalling. We have previously reported a role for PLEKHG5/RAB26 in the regulation of autophagy^9^. Here, we describe an impairment of autolysosomal biogenesis as a result of PLEKHG5-dependent effects at the autophagosome and lysosome fusion in U251-MG cells. Our findings point to a role for PLEKHG5 as a crucial regulator in the autophagic signalling pathway. The knockout of *PLEKHG5* led to a decrease of LAMP-1 protein and thereby disturbance of the lysosomal membrane homeostasis. This might explain the impaired clearance of autolysosomes observed. In line with the literature, both major compartments of the lysosomal membrane LAMP-1 and LAMP-2 are usually regulated differently. Whereas it was published that LAMP-1 is constitutively expressed, the specific LAMP-2 expression is known to vary greatly with the cell type^50^. Moreover, the *LAMP-1* gene was recently shown to be overexpressed in malignant gliomas^25^. Machado and colleagues also described that lysosomal exocytosis, which is also mainly mediated by LAMP-1, plays a primary role in tumour progression and chemoresistance^51^. Although the specific role of LAMP-1 in glioblastoma remains unknown, our results suggest a negative impact of PLEKHG5 depletion followed specifically by a decreased LAMP-1 expression and impaired autolysosome formation on tumour cell survival. In contrast to LAMP-1, expression of LAMP-2 is not affected by PLEKHG5. Furthermore, our study displays that the affection of the cytoskeleton is related to another GTPase for PLEKHG5, namely RhoA^5^. We could demonstrate that the reorganisation of the cytoskeleton is caused by a downregulation of the specific RhoA activity, which could completely be rescued by an overexpression of RAB26 (Fig. 5). According to the literature, RhoA was published to mediate microtubule-dependent cell polarisation and directed cell migration of human brain cancer cells^5^. Similar effects could be observed also from Tseliou and colleagues, whereby a depletion of RhoA was discovered to result in significant alterations in the morphology of cultured glioblastoma cells^52^. Next, we analysed the protein–protein interactions of PLEKHG5 and human proteins by using the bioinformatical online tool STRING^53^, which showed an interaction of PLEKHG5 (red sphere) with various G-proteins, such as RHOA, RHOB and RHOC (Fig. S8A). Interestingly, the here described novel interaction with RAB26, is not included in the previously published data (Fig. S8A). We further analysed accordingly the published interactions with the autophagy marker protein LAMP-1 (Fig. S8B, red sphere). Of note, an interaction of LAMP-1 with the G-protein RAB5C was deduced from curated databases (Fig. S8B), leading to the conclusion that principally an interaction of G-proteins with LAMP-1 is possible. In this context, it is published that LAMP-1 is localised on the lysosome, whereas RAB26 was described to interact directly with the autophagic pathway^43,54^. Thus, it would be interesting to investigate within following studies, if a direct interaction is possible after fusion of the lysosome and autophagosome to the autolysosome. Our data also help to further dissect the interplay between PLEKHG5/RAB26 signalling, NF-κB activation and the regulation of apoptosis. Here, we show that the expression of PLEKHG5 is directly correlated with the specific NF-κB activity. At least in U251-MG cells, the relation between NF-κB activation and apoptosis appears complex. When measuring cleaved Caspase-3, we could only detect an increase in U251-MG rescued cells overexpressing RAB26 (see Fig. 6B). Of note, Caspase-3 is declared as a marker for late apoptotic stages and its activation can remain up to three days^45^, which suggests that the activation of Caspase-3 in *PLEKHG5*^+/+^ , PLEKHG5^−/−^, and PLEKHG5^+/+^ + RAB26QL cells, seemed to be regulated on a time point later than 90 min of TNFα treatment (see Fig. 6B). Thus, we additionally analysed pycnotic nuclei via DAPI staining, whereas the detection of an early stage of apoptosis is described as the specific Annexin V positivity and is published to be directly correlated to the nuclear condensation, which we observed also in our study (see Fig. 6F)^46^. Therefore, we conclude that early stages of apoptosis are regulated by the activity of the PLEKHG5 system. It appears that PLEKHG5 signalling prominently impacts on tumour cell proliferation, possibly also migration^7^, autophagy and apoptosis, all of which have been implied to play a role in the sensitivity of tumour cells to chemotherapy. In summary, cellular fitness might be regulated by a PLEKHG5/RAB26 axis. However, after analysing the direct link from PLEKHG5 to NF-κB activation, we examined some discrepancies between the control levels of luciferase activities (Fig. 7B) and the base levels of the nuclear RELA (Fig. 6D) in U251-MG cells. These differences might be explained with the variation of the experimental trial but nonetheless represent the limitations of this study. Furthermore, some limitations of this study are the usage of U251-MG cells and derivate cells as a model system for the heterogeneous glioblastoma multiforme. Hence we investigated the most important chemotherapy paradigm for glioblastomas, the alkylating agent TMZ against the background of variable *MGMT* promoter methylation presumably resulting in epigenetic silencing^2^. Importantly, MGMT is a downstream target of the NF-κB pathway^10^. Our analysis revealed a complex picture. All GBM cells investigated proved not very sensitive to TMZ at the time and concentrations tested in our study. Interestingly, U251-MG cells with the lowest degree of *MGMT* promoter methylation (*PLEKHG5*^−/−^) have the lowest cellular fitness. The very substantial increase of MGMT expression upon transduction with RAB26QL did not render cells chemoresistant when compared to the wildtype U251-MG cells.

In conclusion, we have analysed the role of PLEKHG5/RAB26 signalling in a GBM cell culture model in considerable detail (Fig. 8D). Our results reveal a prominent regulatory function of PLEKHG5 in the control of the NF-κB activation. We provide substantial evidence that the PLEKHG5/RAB26 pathway is involved in the regulation of autophagy, cell proliferation, cellular morphology, apoptosis and even MGMT expression as well as promoter regulation at least in the U251-MG glioblastoma model system under investigation. In particular, the markedly reduced proliferative properties, the affection of the cell morphology and the reduced cellular viability of PLEKHG5-deficient cells indicate that interference with this complex molecular network might turn out to be a promising therapeutic strategy for other glioma cell lines and further in primary GBM.

## Material and methods

### Target design and cloning

Using the online forecast tool from the University of Heidelberg, the single guide RNAs (sgRNAs) were designed in silico for further practical usage^55^. For minimisation of putative off-target effects, the Cas9n (D10A) nickase mutant was chosen with appropriating nicking pairs^35^. Two nicking pairs were selected for the generation of a gene knockout, whereas here the deletion was spanning an intron–exon boundary. Designed sgRNAs were cloned into pSpCas9n(BB)-2A-Puro (PX462) V2.0 (plasmid #62987, Addgene, Teddington, UK) as already published^35^. Oligo-sequences for used sgRNAs:

oligo 1: (fwd 5′-CACCGACAGCACCATGCATTATGA-3′;
  rev 5′-AAACTCATAATGCATGGTGCTGTC-3′).
oligo 2: (fwd 5′-CACCgGCTGTCACAGGCCTCGCAG-3′;
  rev 5′-AAACCTGCGAGGCCTGTGACAGCC-3′).
oligo 3: (fwd 5′-CACCgGGGAGAGGGGGGACTGCTG-3′;
  rev 5′-AAACCAGCAGTCCCCCCTCTCCCC-3′).
oligo 4: (fwd 5′-CACCgCATCAAGTTTCCCCTCTGC-3′;
  rev 5′-AAACGCAGAGGGGAAACTTGATGC-3′).

### Cell culture and transfection of U251-MG

Human embryonic kidney (HEK) 293FT cells, HeLa cells from human cervix adenocarcinoma and human U251-MG cells were cultivated in growth media, containing DMEM (Life Technologies, Darmstadt, Germany), 10% fetal calf serum (Life Technologies), penicillin/streptomycin (50 mg/ 5 ml; PAA Laboratories, Linz, Austria) and L-glutamine (200 mM, PAA Laboratories) at 37 °C and 5% CO_2_ in a humidified incubator. For the CRISPR/Cas9-mediated knockout, U251-MG cells were transfected by using TurboFect transfection reagent (Thermo Fischer Scientific, Dreieich, Germany), according to manufacturer’s guidelines. Potential *PLEKHG5* knockout cells were clonally grown from previously separated and seeded single cells per 0.3 cm^2^, whereas the clone H5.1 was further used for all experiments of the study.

### Proliferation, cell viability and apoptosis assays

To analyse the proliferative behaviour of U251-MG cells via determination of the population doubling time, 50,000 cells per 9 cm^2^ were seeded and cultivated for 6 h or 48 h. Subsequently, cells were harvested and cell number was determined. Accordingly to manufacturer’s guidelines, 5000 cells per 0.3 cm^2^ were seeded and incubated for 48 h in growth media, partially added with different concentrations of TMZ (Sigma-Aldrich, Taufkirchen, Germany). To determine the cellular viability, the Orangu Cell Proliferation Assay Kit (Cell Guidance Systems Ltd, Cambridge, UK) was used. Cellular viability of *PLEKHG5* wildtype was normalised to their control condition. For PLEKHG5-deficient as well as rescue cells and U251-MG wildtype with constitutive RAB26 expression of the respective condition, cellular viabilities were displayed in percentage, normalised to the *PLEKHG5* wildtype. Apoptotic cells were identified using cleaved Caspase-3 immunocytochemistry and DAPI staining of the nuclei (see 4.8), which was analysed via determination of the signal intensity, nuclear fragmentation and size. For examination of the average percentage of apoptotic cells, the ratio of cleaved Caspase-3 positive cells or the number of apoptotic cells per exemplary picture, were examined to the total amount of analysed cells. Quantification as well as statistical analysis were performed via ImageJ software (NIH, USA) and Prism V5.01 software (GraphPad Software, Inc., California, USA).

### Transfection of HEK 293FT cells for lentivirus production

To produce lentiviral vectors, HEK 293FT cells were seeded by the density of 2 × 10^6^ cells per 75 cm^2^ in culture dishes with growth media for 48 h at 37 °C. Afterwards, cells were treated with 1 ml calcium-phosphate precipitate, containing 6 µg of pCMV-VSV-G envelope plasmid (Addgene), 15 µg of the pCMV-ΔR8.91 (Addgene) vector, to encode the virion proteins and 20 µg of cFUGW-RFP-GFP-LC3^+^, cFUGW-EGFP-RAB26-WT or cFUGW-EGFP-RAB26QL^9,54^ (vectors were kindly provided by the Sendtner-lab, Würzburg, Germany). cFUGW-RFP-GFP-LC3^+^ served as reporter system, cFUGW-EGFP-RAB26QL as the constitutive active variant and cFUGW-EGFP-RAB26-WT as a transduction control gene. Plasmids were dissolved, 500 µl 2 × HBS and 50 µl 2.5 M CaCl_2_ were added, followed by incubation for 20–25 min at room temperature before transfection. Medium was changed after cultivation of 6 h at 37 °C. To isolate the lentivirus, medium of each type was collected and span down after two more days of cultivation. The supernatants were filtered and ultra-centrifuged for 2 h with 178,100 g at 4 °C. The formed pellets comprising lentiviral plasmids, were resuspended in 1 ml serum-free media and stored at − 20 °C.

### Lentiviral transduction of U251-MG cells

To transduce glioma cells with RAB26QL and mRFP-GFP-LC3^+^ reporter constructs, 20,000 cells per 2 cm^2^ in a 24-well plate were seeded in growth medium, including 8 µl polybrene (10 mg/ml, Sigma-Aldrich) and 60 µl of the concentrated lentivirus plasmids. After three times of passaging, the efficiency of transduction was assessed with fluorescence imaging and further analysis were performed using a confocal laser-scanning microscope (LSM 510 and LSM 780, Carl Zeiss, Jena, Germany).

### Genomic PCR

1 × 10^6^ cells from U251-MG cell lines were pooled and resuspended in 300 µl lysis-buffer with SDS as well as 200 ng/ml Proteinase K and shook for 4 h at 55 °C. After a heat shock with 95 °C for 5 min, the genomic PCR was performed with GoTaq DNA polymerase (Promega Corporation, Mannheim, Germany) according to manufacturer’s guidelines. Sequences for used PLEKHG5 primer pair:

PLEKHG5: (fwd 5′-TTGTCCTTATGACGCCCTAGC-3′;
  rev 5′-CACTGCACTCCCTGTCTCAAAGAA-3′).

Amplified DNA was loaded on a 2% agarose gel with 0.001% EtBr (Carl Roth GmbH, Karlsruhe, Germany) and ran for 30 min by 100 V, followed by imaging with a trans-illuminator (UVsolo TS, Biometra).

### RT-PCR and quantitative RT-PCR

RNA from 1 × 10^6^ cultured and pooled U251-MG cells were isolated by using the TRI Reagent protocol (Sigma-Aldrich) according to manufacturer’s guidelines. To remove interfering DNA, DNase digestions were performed afterwards, according to the guidelines of Thermo Fisher Scientific. RNAs were transcribed into cDNA according to the protocol from Promega Corporation and Thermo Fisher Scientific for cDNA synthesis. Non-quantitative analysis on mRNA level was performed via *One*Taq polymerase (New England Biolabs, Frankfurt am Main, Germany) according to manufacturer’s guidelines. For quantitative RT-PCR, the qPCR SYBR Green Mix (PCR BIOSYSTEMS, London, UK) as well as the Eco48 cycler from PCRmax (Staffordshire, UK) were used according to their protocols. Sequences used for RAB26 primer pair:

RAB26: (fwd 5′-TGCGATTCAAGGATGGTGCT-3′;
  rev 5′-CACACCATCCACGTCCAGAA-3′).

After non-quantitative RT-PCR, amplified transcripts were loaded on a 3% agarose gel with 0.001% EtBr and ran for 50 min by 100 V, subsequently imaged with a trans-illuminator (Biometra). Data evaluation of qRT-PCR results was performed with the related ProStudy software from PCRmax (Staffordshire).

### Immunocytochemical staining

For immunocytochemical staining, cells were seeded at the top of sterilised and etched coverslips with 20,000 cells per 4 cm^2^ in 12-well plates with 1 ml growth medium and cultivated for 48 h. To analyse the localisation of RELA signals as an indication of the NF-κB activity or to determine the apoptosis via cleaved Caspase-3, U251-MG cells were stimulated with TNFα (final concentration 10 ng/ml, Merck, Darmstadt, Germany). After different treatment periods, cells were fixed with 4% paraformaldehyde in PBS for 10 min and washed three times with PBS. Blocking of free binding sides and permeabilisation were done with PBT solution, including 0.02% Triton-X-100 (Sigma-Aldrich) and 5% goat serum (DIANOVA, Hamburg, Germany) in PBS for 30 min. Afterwards, three washing steps with PBS were followed by an incubation with primary antibodies (cleaved Caspase-3 and NF-κB p65 (RELA), dilution 1:400; Cell Signaling, Munich, Germany; MGMT, dilution 1:50, Merck; PLEKHG5, dilution 1:100, Abnova, Taipei City, Taiwan; RAB26, dilution 1:200, Synaptic Systems, Göttingen, Germany). After further washing steps, the secondary fluorochrome-conjugated antibody (Alexa 555, diluted 1:300 in PBS, Life Technologies) was applied even for 1 h under exclusion of light. For nuclear and cytoskeleton staining, DAPI (0.5 µg/ml, Sigma-Aldrich) and phalloidin/rhodamine (0.1% v/v, Sigma-Aldrich) were applied for 10 min. Before the cells were embedded in Mowiol 4-88 (Carl Roth GmbH) upside down on the top of a microscope slide, another washing step was performed. For fluorescence imaging, the confocal laser-scanning microscope (LSM 780, Carl Zeiss) was used. Measurement of the cell area via phalloidin/rhodamine and localisation of RELA as well as cleaved Caspase-3 signals were analysed via ImageJ software (NIH) and Prism V5.01 software (GraphPad Software).

### SDS-PAGE and immunoblotting

For preparation of whole cell lysates, cells were washed with PBS and partially treated with 200 nM Bafilomycin A1 (TOCRIS, Bristol, UK) for 4 h in growth media. Cells were harvested, resuspended in 1 ml PBS and centrifuged for 1 min at 11,500 *g*, followed by discarding the supernatant and resuspension of the pellet in 300 µl of a 1:5 diluted passive luc-lysis buffer (Promega Corporation) in PBS. The proteinase inhibitors Pepstatin A (1 µg/ml, Sigma-Aldrich), Leupeptin Hemisulfate (10 µg/ml, Carl Roth GmbH) and Aprotinin (1 mg/ml, Carl Roth GmbH) were also added. Suspension was treated 30 secs with an ultrasonic rod and centrifuged for 1 min at 11,500 *g*. The average amounts of protein were measured and 10 µg protein of each condition were separated with loading buffer and heated for 5 min at 95 °C. Lysates were loaded onto a denaturating SDS–polyacrylamide gel for electrophoresis, whereas the gels were running at 15 mA for 1 h. Proteins were transferred to a Roti-PVDF membrane (Carl Roth GmbH) with a semi-dry blotter for 1.5 h with 130 mA per gel. Afterwards membranes were washed 10 min with PBS, including 0.05% Tween-20 (VWR International GmbH, Darmstadt, Germany) and blocked 1 h at 37 °C in 5% powdered milk in PBS (Carl Roth GmbH). Binding of the membrane loaded proteins to first antibodies, diluted in blocking solution were performed at least over night at 4 °C (β-Actin, Calnexin, LAMP-1 and LAMP-2, dilution 1:1000; all from Cell Signaling; PLEKHG5, dilution 1:1000, Abnova, Taipei City, Taiwan and RhoA, dilution 1:670, Thermo Fisher Scientific). After incubation, three additional washing steps were performed and the secondary HRP-conjugated antibodies (mouse/rabbit, diluted 1:4000 in blocking solution, DIANOVA) were applied for 1 h at 25 °C. Before the amounts of proteins were measured, membranes were washed with PBST and placed in an x-ray film cassette for further light protection. Results were visualised by enhanced chemiluminescence with a solution containing 1 ml of Solution A (Sigma-Aldrich), 0.3 µl 30% H_2_O_2_ and 100 µl Solution B (Sigma-Aldrich), for each membrane. A radiographic film (Super RX-N, FUJIFILM, Düsseldorf, Germany) was carefully served for chemiluminescent detection. The different protein amounts were normalised to their related Calnexin or β-Actin signals, which functioned as loading controls. Quantification and statistical analysis were performed by using ImageJ software (NIH) and Prism V5.01 software (GraphPad Software).

### Transient transfection of U251-MG cells and luciferase activity assay

For transient transfection, U251-MG cells were cultured, harvested and 1 × 10^6^ cells per cell line were co-transfected with TK(NF-κB)_6_LUC vector (1 µg,^56^) and pRLcmv vector (2 µg, Promega Corporation), using the Nucleofector II device (Lonza Group, Basel, Switzerland) via program T-027 and Cell Line Nucleofector Kit T (Lonza Group), according to manufacturer’s guidelines. PmaxGFP (Lonza Group) served as transfection control. After transient transfection, cells were firstly cultivated for 48 h in growth medium and afterwards treated for further 24 h with stimulation medium, containing TNFα (final concentration 10 ng/ml, Merck). Subsequently, analysis of NF-κB activation in U251-MG cells were performed via assessment of luciferase activities, by using the Dual-Luciferase Reporter Assay System Kit and the GloMax-Multi + Detection System, according to the guidelines of Promega Corporation. All statistical analysis were performed via Prism V5.01 software (GraphPad Software). Ratios of the dual luciferase reporter assay were determined via normalisation of the *firefly* luciferase activity to the corresponding amount of the *Renilla* activity. Subsequently, fold changes for the different cell lines and conditions were examined in relation to the untreated U251-MG *PLEKHG5* wildtype.

### Rho GST-RBD assay

To assess specific RhoA activities in U251-MG cell lines, different GST-RBD pulldown assays were performed by using the Pierce Active GTPase Pulldown- and Detection-Kit (Thermo Fischer Scientific), according to manufacturer’s guidelines. Prepared lysates were loaded onto a denaturating SDS–polyacrylamide gel for electrophoresis and were further analysed via Western blotting, as described in the manual of Thermo Fisher Scientific. Chemiluminescent signals from active (RBD-bound) RhoA were normalised to their respective protein amounts of total RhoA in whole cell lysates, as it was published by Basak and colleagues^57^. Quantification and statistical analysis were performed via ImageJ software (NIH) and Prism V5.01 software (GraphPad Software).

### Promoter methylation analysis

To determine the *MGMT* promoter methylation status, genomic DNA from U251-MG cell lines (see 4.6 for isolation protocol) were analysed in the Department of Neuropathology, at the University Hospital Erlangen, Germany. According to manufacturer’s guidelines, samples were firstly bisulfide converted by using the EpiTect Bisulfite Kit (Qiagen, Hilden, Germany) and the prescribed sequence of interest was afterwards sequenced via pyro-sequencing with the *therascreen* MGMT Pyro Kit (Qiagen).

Sequence used for analysing:

- MGMT: 5′-C/TGTTTTGC/TGTTTC/TGAC/TGTTC/TGTAGGTTTTC/TGC/TG-3′.

### Statistical analysis and figure design

Data were analysed by the usage of Prism V5.01 software (GraphPad Software). Test for normality was performed via D’Agnostino & Pearson omnibus normality test. Non-parametric one-way ANOVA with Bonferroni's multiple comparison test, two-way ANOVA with Bonferroni post-test and Mann–Whitney U test or paired and unpaired t tests were performed to assess differences between multiple groups. For all analysis, a *p* ≤ 0.05 value was considered as statistically significant. Evaluated data are displayed as the means ± standard error of the mean (SEM). All figures were designed by using the CorelDRAW Graphics Suite 2018 software (Corel Corporation, Ottawa, Canada).

## Supplementary information

Supplementary information.
