# Supplementary material for: PLEKHG5 regulates autophagy, survival and MGMT expression in U251-MG glioblastoma cells
Source: Sci Rep. 2020 Dec 14;10:21858. doi: 10.1038/s41598-020-77958-3 (PMC7736842; doi:10.1038/s41598-020-77958-3)
Supplement: Supplementary file 1 — Supplementary information. [file 41598_2020_77958_MOESM1_ESM.pdf]

PLEKHG5 regulates autophagy, survival and MGMT expression in U251-MG glioblastoma cells

Kaya Elisa Witte[1,2,5*], Carsten Slotta[1,2], Melanie Lütkemeyer[1], Angelika Kitke[1], Roland Coras[3], Matthias Simon[4,5], Christian Kaltschmidt[1,5+] and Barbara Kaltschmidt[1,2,5+*]

[1] Department of Cell Biology, University of Bielefeld, Universitätsstr. 25, 33615 Bielefeld, Germany
[2] Molecular Neurobiology, University of Bielefeld, Universitätsstr. 25, 33615 Bielefeld, Germany
[3] Department of Neuropathology, University Hospital Erlangen, Schwabachanlage 6, 91054 Erlangen, Germany
[4] Department of Neurosurgery, Protestant Hospital of Bethel Foundation, Burgsteig 13, 33617 Bielefeld, Germany
[5] Research Association of BioMedicine Bielefeld, FBMB, Maraweg 21, 33617 Bielefeld, Germany

[+] These authors contributed equally to this work
* Corresponding Authors:
Barbara Kaltschmidt, Department of Cell Biology, University of Bielefeld, Universitätsstr. 25, 33615 Bielefeld, Germany, Tel.: + 49 521 106 5624; Fax: + 49 521 106 5654; E-Mail: barbara.kaltschmidt@uni-bielefeld.de
Kaya Elisa Witte, Department of Cell Biology, University of Bielefeld, Universitätsstr. 25, 33615 Bielefeld, Germany, Tel.: + 49 521 106 5629; Fax: + 49 521 106 5654; E-Mail: kaya.friedrich@uni-bielefeld.de

Description of Supplementary Files

File Name: Supplementary Informations
Description: Supplementary Figures

Supplementary Figure S1

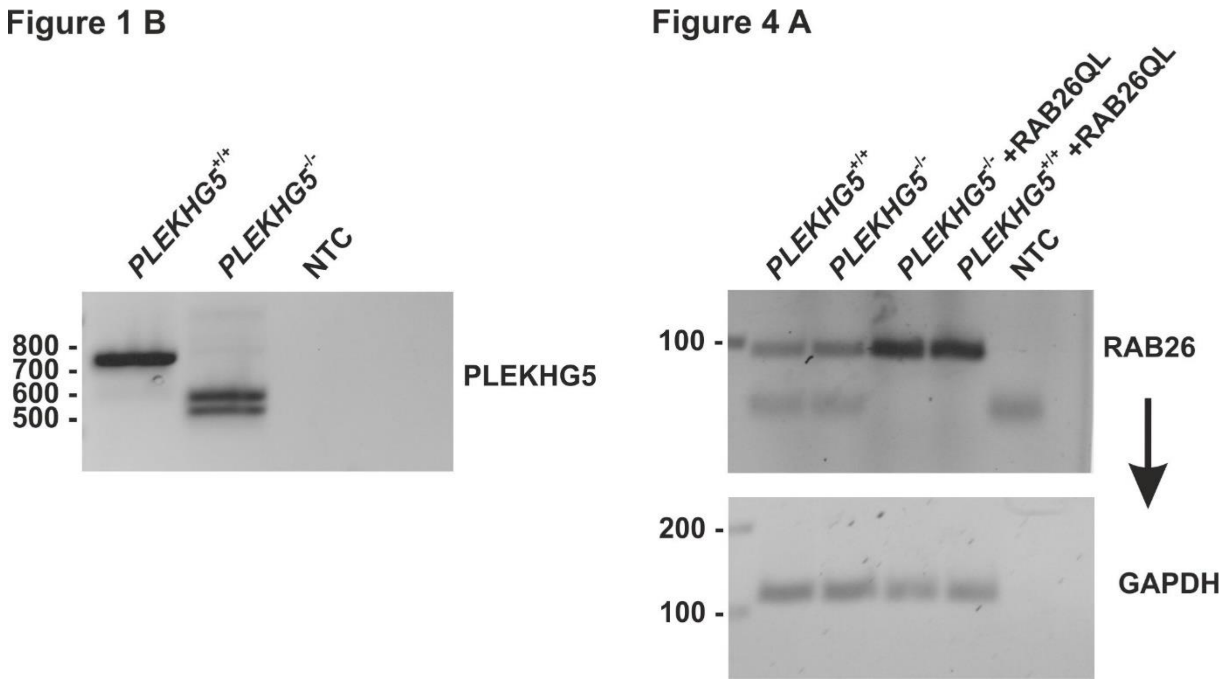

Full size images of RT-PCRs.
Arrow indicates the reutilisation of cDNA probes.

Supplementary Figure S2

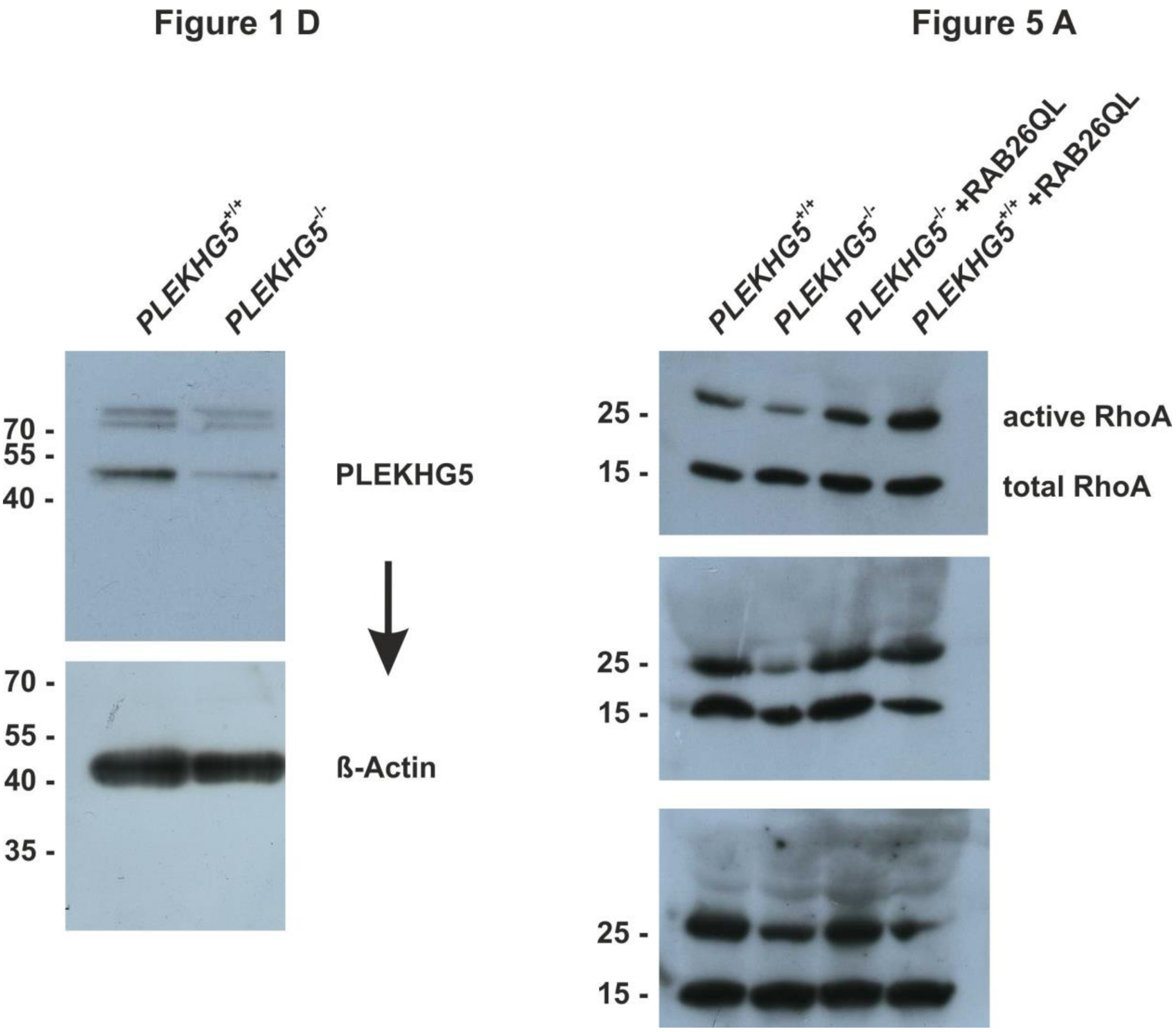

Full size images of Western blots for PLEKHG5 detection and RBD-bound RhoA quantification.

The arrow indicates reprobing of the Western blot for PLEKHG5 detection with ß-Actin as a loading control. For RhoA expression, lower bands represent total RhoA amounts, whereas upper bands display the active form of the protein.

Supplementary Figure S3

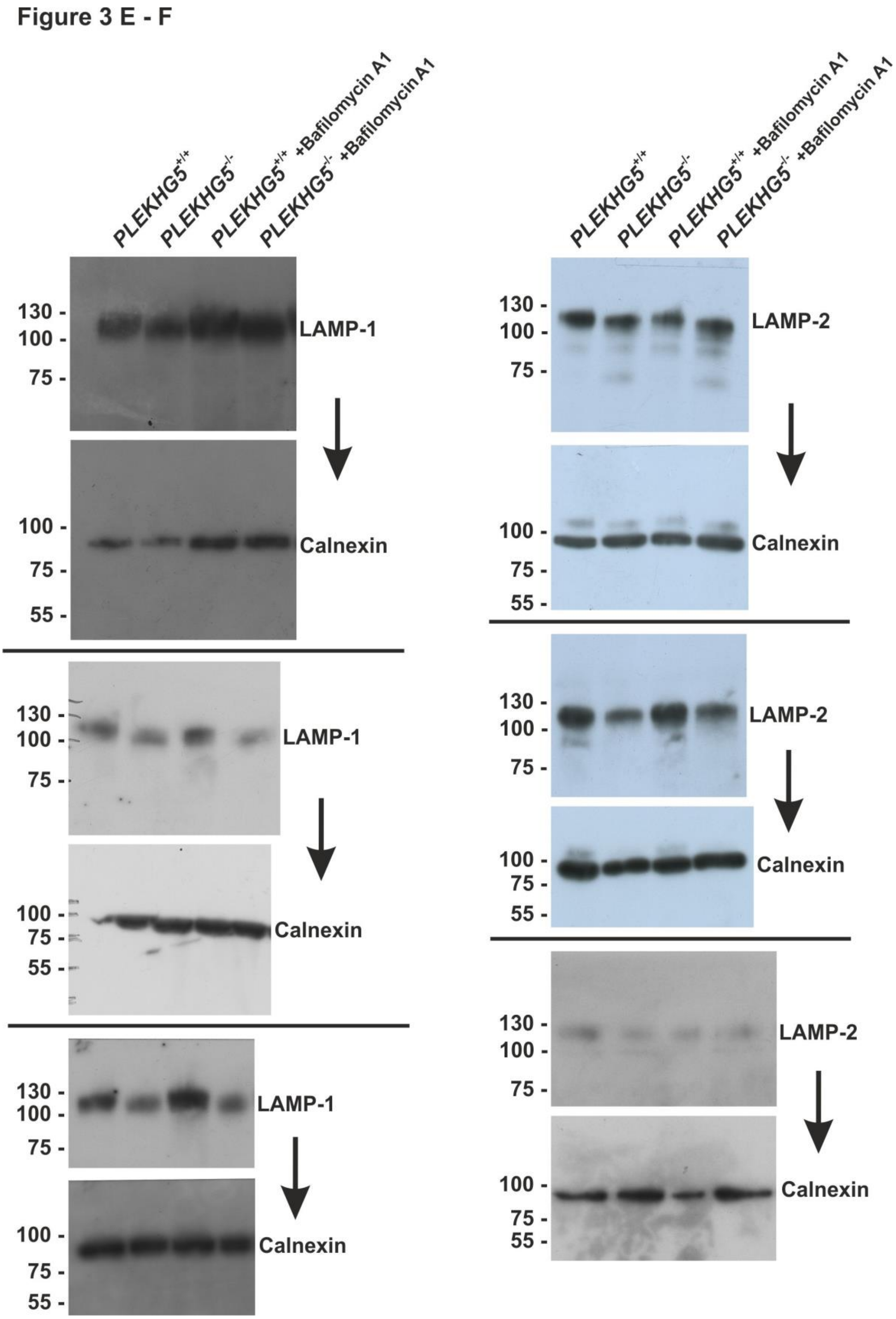

Full size images of Western blots for quantification of LAMP-1 and LAMP-2.
Arrows indicate reprobing of Western blots. Lines separate the individual blots.

Supplementary Figure S4

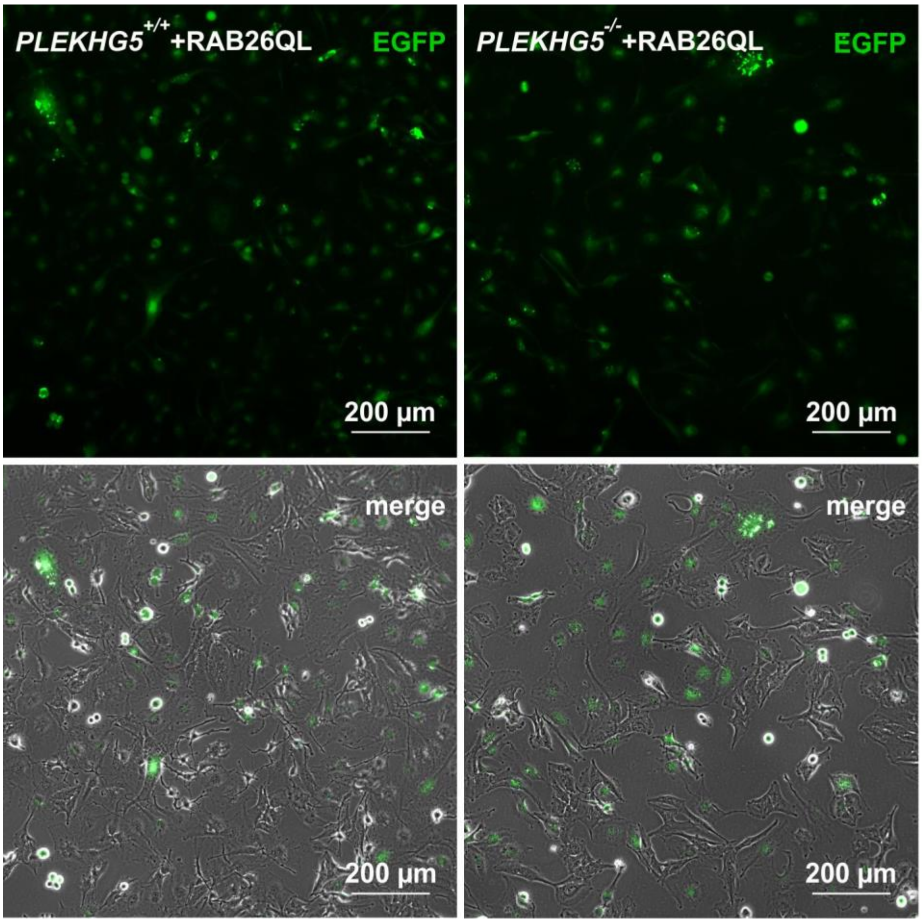

Fluorescent tracing of constitutively active RAB26QL, as a lentivirus-mediated transduction control.

Visualisation of positive transduced U251-MG cells, by lentiviral transduction with FUGW-EGFP-RAB26QL plasmids, depicted with EGFP as fluorescent tracing.

Supplementary Figure S5

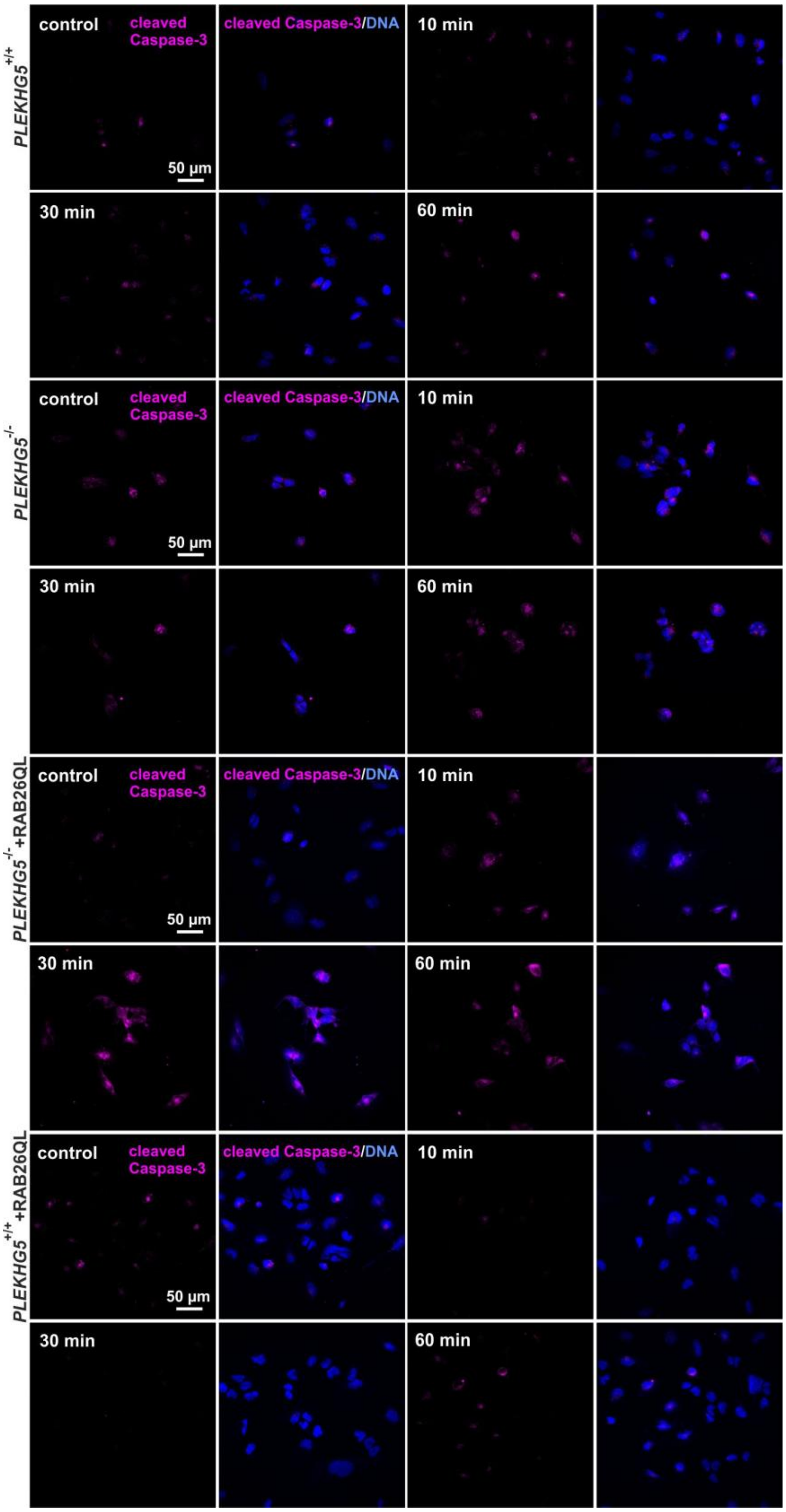

Immunocytochemistry of U251-MG cells depicting cleaved Caspase-3 after partial stimulation with TNFα.

Exemplary pictures of U251-MG cell lines treated with various time sections of TNFα and untreated controls.

Supplementary Figure S6

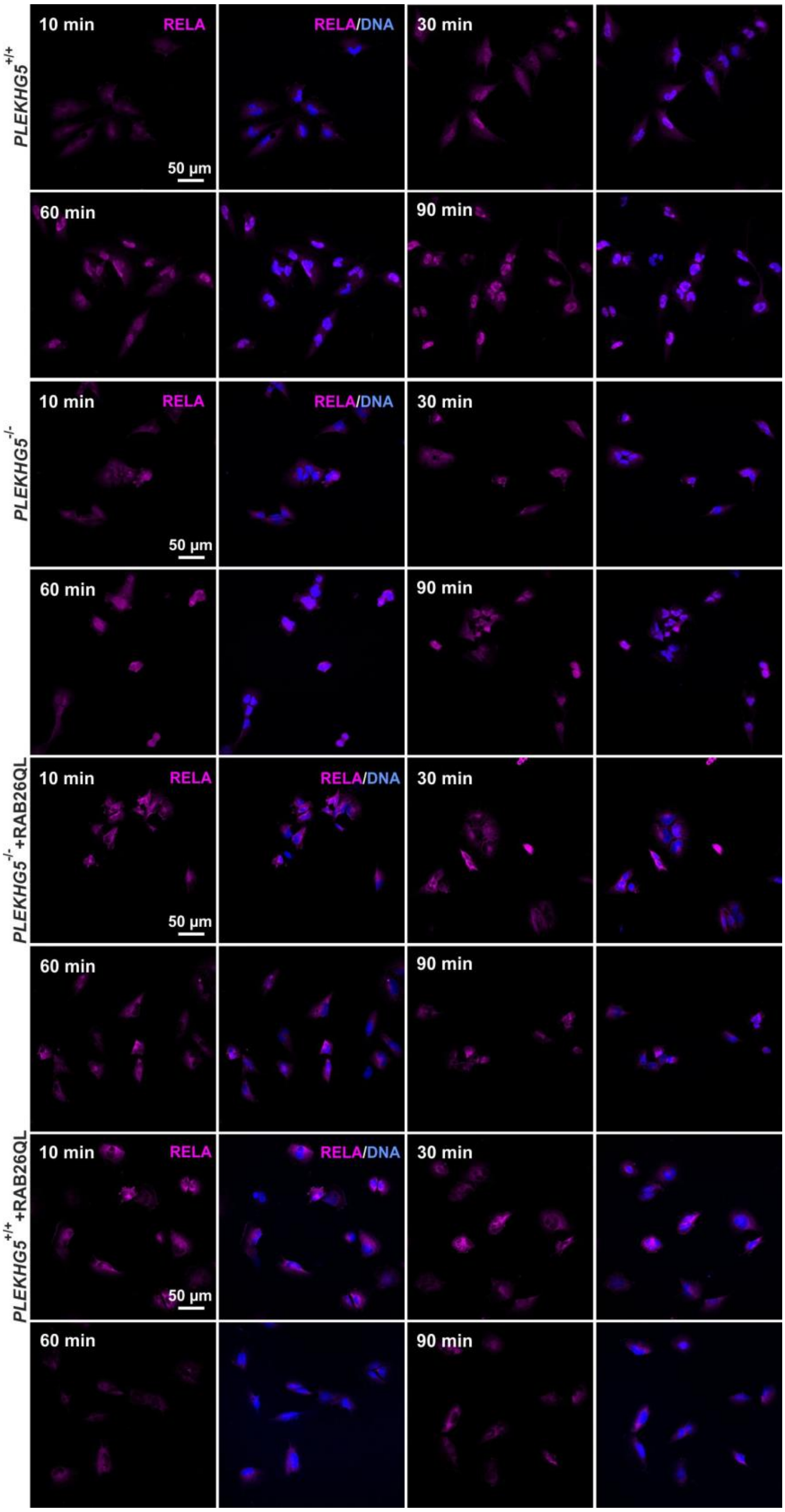

Immunocytochemistry of RELA expression in U251-MG cells after TNFα stimulation. Exemplary pictures of U251-MG cell lines treated with various time sections of TNFα and untreated controls.

Supplementary Figure S7

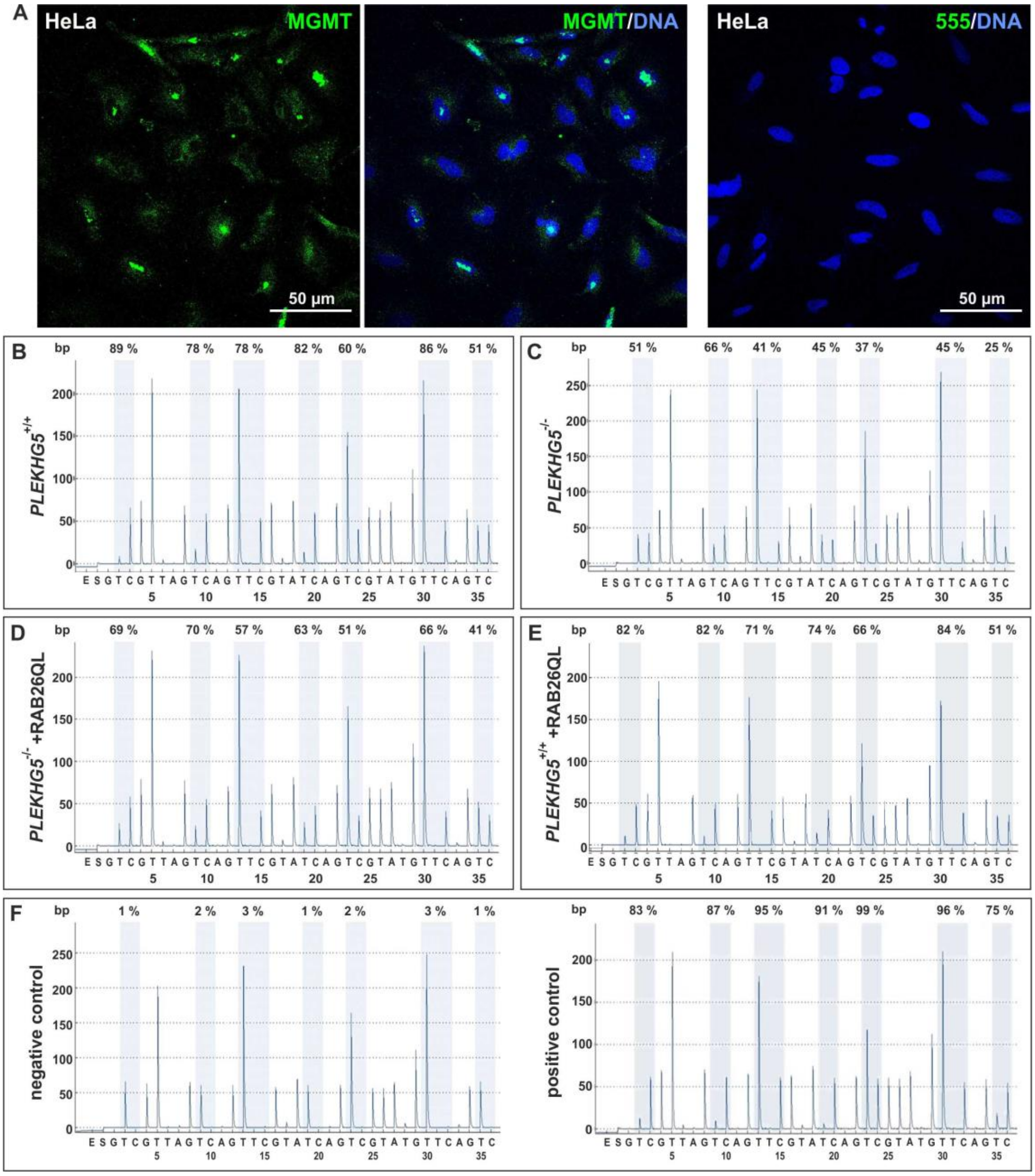

Immunocytochemistry of MGMT in HeLa cells and graphical analysis of the *MGMT* promoter methylation status.

A: Immunocytochemistry of cultured HeLa cells depicting MGMT expressions. Right picture was performed as a negative control, incubated without the specific primary MGMT antibody. **B - E:** U251-MG cell lines were analysed for their promoter methylation on a prescribed *MGMT* sequence according to the therascreen MGMT Pyro Kit, in relation to the amount of methylated base pairs. **F:** Promoter methylation analysis of technical standards. Whereby the negative control (left graph) depicted a range of 1 % - 3 % methylated CpG-islands, the positive control (right graph) showed a *MGMT* methylation from 75 % up to 99 %.

Supplementary Figure S8

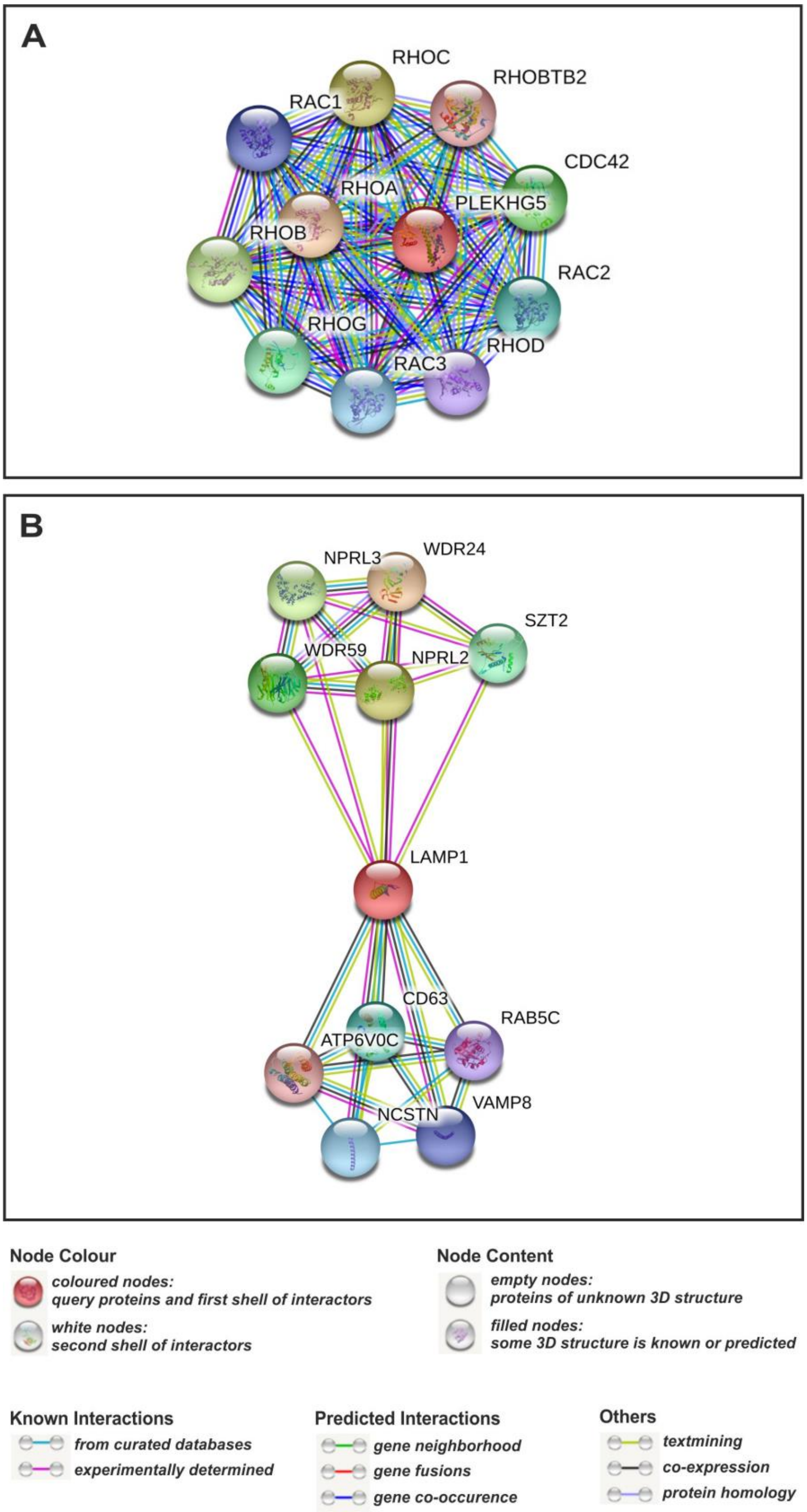

Interactome analysis of PLEKHG5 and LAMP-1.

A: Protein-protein interactions of PLEKHG5 to published G-proteins. **B:** Analysis of interactions between LAMP-1 and previously described human proteins like RAB5C. Data curation by STRING [51].
